# S-nitrosylation of IRF7 induced by NOS1 expression in melanoma suppresses anti-tumor immunity

**DOI:** 10.1038/s41419-025-08201-y

**Published:** 2026-01-14

**Authors:** Yuxin Dai, Sisi Zeng, Keyi Li, Jinyan Huang, Minzhu Yang, Wenwen Gao, Xi Chen, Mengqiu Huang, Shuangyan Ye, Qianli Wang, Jianping Chen, Lingqun Zhu, Zhuo Zhong, Shuai Yuan, Siwei Guo, Nan Liu, Bingtao Hao, Qiuzhen Liu

**Affiliations:** 1 Cancer Research Institute, School of Basic Medical Science, Southern Medical University, Guangzhou, China; 2 Department of Pathology, Nanfang Hospital, Southern Medical University, Guangzhou, Guangdong China; 3 Department of Pathology, School of Basic Medical Sciences, Southern Medical University, Guangzhou, Guangdong China; 4 Guangdong Province Key Laboratory of Molecular Tumor Pathology, Guangzhou, Guangdong China; 5 Pingshan District People’s Hospital of Shenzhen, Pingshan General Hospital of Southern Medical University, Shenzhen, Guangdong China; 6 First Affiliated Hospital of Zhengzhou University, Zhengzhou, Henan China; 7 Zhongshan Torch Development Zone People’s Hospital, Zhongshan, Guangzhou China; 8 Department of Oncology, The Sixth Affiliated Hospital, School of Medicine, South China University of Technology, Foshan, Guangdong China; 9 Zhujiang Hospital of Southern Medical University, Guangzhou, China; 10 Department of Medical Oncology, Affiliated Cancer Hospital of Zhengzhou University, Henan Cancer Hospital, Zhengzhou, China; 11 Department of Radiotherapy, The Affiliated Qingyuan Hospital (Qingyuan People’s Hospital), Guangzhou Medical University, Qingyuan, PR China; 12 Radiotherapy Department Guangzhou Concord Cancer Center (GCCC) No. 9 Ciji Road (Sino-Singapore Guangzhou Knowledge City), Guangzhou, China; 13 Oncology Department, Guangzhou Hospital of Integrated Traditional and West Medicine, Guangzhou, China; 14 Department of Obstetrics and Gynecology, Nanfang Hospital, Southern Medical University, Guangzhou, China; 15 Department of Immunology, School of Basic Medical Sciences, Zhengzhou University, Zhengzhou, Henan China; 16 Henan Eye Institute, Henan Academy of Innovations in Medical Science, Zhengzhou, Henan China

**Keywords:** Cancer microenvironment, Melanoma, Melanoma

## Abstract

Endogenous nitric oxide (NO) produced by nitric oxide synthases (NOSs) plays an important immunosuppressive role in the tumor microenvironment. In melanoma, NOS1 expression increases with tumor progression and correlates with tumor immune escape through the inhibition of type I interferon (IFN) signaling. However, the immune regulatory role and related mechanisms of NOS1, as well as its impacts on immune therapies such as immune checkpoint blockade (ICB) in melanoma, remain unclear. Here, we found that NOS1 expression induces IRF7 modification by S-nitrosylation at the C435 site in mice (C481 in humans), which functionally promoted tumor growth in mouse models. Mechanistically, IRF7-C435-SNO inhibited IFNβ transcription under PRR signal activation, leading to a disorder in the initiation of the type I interferon response in melanoma cells. In a melanoma mouse model, IRF7-C435-SNO decreased the infiltration and activation of CD8 + T cells in the tumor microenvironment by reducing antigen presentation processes in tumor cells and inhibiting the maturation of DC1. Clinically, high expression of NOS1 correlated with poor survival prognosis and resistance to ICB anti-tumor therapies in melanoma cases with less immune cell infiltration. Our study suggests that NOS1 expression in melanoma characterizes IFN-I signal disorders in response to innate immune stimulation through IRF7 s-nitrosylation. Targeting NOS1 signaling might be beneficial for overcoming immune therapeutically resistance, particularly in immune-cold melanoma phenotype.

## Introduction

Cutaneous melanoma is considered one of the most immunogenic tumor types and benefits clinically from immunotherapy such as immune checkpoint blockade (ICB). However, the majority of patients exhibit primary or required resistance to this treatment strategy [1–4]. It has been postulated that tumor intrinsic mechanisms involved in the {Citation}ability of the therapy resistance. A diverse set of mechanisms has been identified, including β-catenin activation, PTEN loss, loss of antigen presentation machinery, impaired interferon responsiveness, all contributing to resistance to immuno-therapy [5–7]. These mechanisms focus on the direct or indirect decrease of antigen expression in tumor cells, enabling immune evasion and promoting the immunosuppressive polarity in the tumor microenvironment [8–10]. During melanoma progression, there is a progressive loss of antigen presentation capacity to cytotoxic T-cells by tumor cells for reducing their immunogenicity, which is essential for T cell activation [10, 11]. Identifying actionable molecules that could be targeted to increase antigen presentation and reverse the immunosuppressive polarity in tumor sites may synergize with immunotherapy to enhance melanoma treatment.

Endogenous NO, synthesized by nitric oxide synthases (NOS1, NOS2 and NOS3), acts as a master regulator of cancer development and progression. NOSs have been found to have increased expression in a variety of tumor types and are involved in the regulation of multiple biological functions necessary for tumor maintenance, growth, immune escape, metastasis, and drug resistance [12–15]. In the tumor microenvironment, cell-derived NO can react with oxygen to form reactive nitrogen species such as N₂O₃ or NO⁺. These NO-derived species mediate post-translational modifications—including S-nitrosylation and tyrosine nitration—on receptors and signaling components on immune cells, thereby modulating tumor immunity. [16–20]. IFN-α, a cytokine that plays a critical role in the regulation of antigen presentation, was the first cancer immunotherapy approved by the FDA. Low response to IFNα, caused by dysfunction of type I interferon signaling, down-regulates the infiltration of immune cells into tumor sites, form an “immunological cold tumor” [21–26]. NO-producing MDSCs in tumor-bearing hosts would inhibit DC antigen presentation by inducing nitration of STAT1-Tyr701 in melanoma patients [27]. Our studies, along with others, have shown that NOS1 expression in melanoma induces dysfunction of type I IFNs signaling [28–30]. However, the mechanisms by which NO derived from tumor cells regulates IFNs still need to be explored in detail.

Interferon (IFN) regulatory factor 7 (IRF7) is a key transcription factor for activation of IFNα signaling in somatic cells in response to pathogens or cellular stress [31]. IRF7, in conjunction with IRF3, activates the transcription of IFN-stimulated genes, as well as small amounts of type 1 IFN (IFNα/β), and the latter leads to additional transcription of IRF7 via activation of the JAK/STAT signaling cascade [32]. This positive feedback loop is essential for the elimination of viral invasion and also has antitumor functions [33, 34]. In a mouse model, IRF7-mediated immunity is critical for preventing metastasis to bone. In breast cancer patients, elevated expression of IRF7-regulated genes in the primary lesion has been associated with prolonged metastasis-free survival [35, 36]. The activation of IRF7 is regulated by post-transcription modifications such as phosphorylation, oxidation, and ubiquitination [37, 38]. The regulation of IRF7 by NO-induced modification has not been reported to our knowledge [39].

Three subtypes of NOSs have been demonstrated to be involved in melanoma progression by promoting melanoma cell proliferation, invasion, resistance to apoptosis and metastasis, with NOS2 correlating with poor patient survival [40]. However, NO signaling might be an important driver for melanoma progression. Melanoma cells transform from melanocytes, which originally express NOS1 under ultraviolet light to promote cellular proliferation [41]. SNP genotypes of NOS1 are associated with an increased risk of melanoma [42]. NOS1 is expressed in 80% of melanomas, and NOS1 activity is higher in melanomas than in melanocytes [43]. Pro-tumorigenic IFN-γ treatment significantly increases nNOS expression levels in melanoma cells. NOS1-selective small molecule inhibitors possess potent anti-melanoma activity in vivo and effectively inhibited the induction of PD-L1 stimulated by IFN-γ treatment [44]. Understanding the specific role of melanoma NOS1 in immune regulation may provide insight into the cellular mechanisms behind the immune resistance during malignant progression. NOS1 produces low levels of NO to induce modification of S-nitrosylation (binding NO to specific cysteine residues) on target proteins [45]. In this study, we investigated the role of NOS1 expression in regulating IRF7 function by S-nitrosylation regulation, and found that NOS1 induces IRF7 S-nitrosylation, which inhibits the activation of type I IFNs signal in melanoma, leading to the formation of an immunosuppressive microenvironment.

## Results

### NOS1 expression inhibits Poly(I:C)-induced IFN-β transcription

**Fig. 1:**
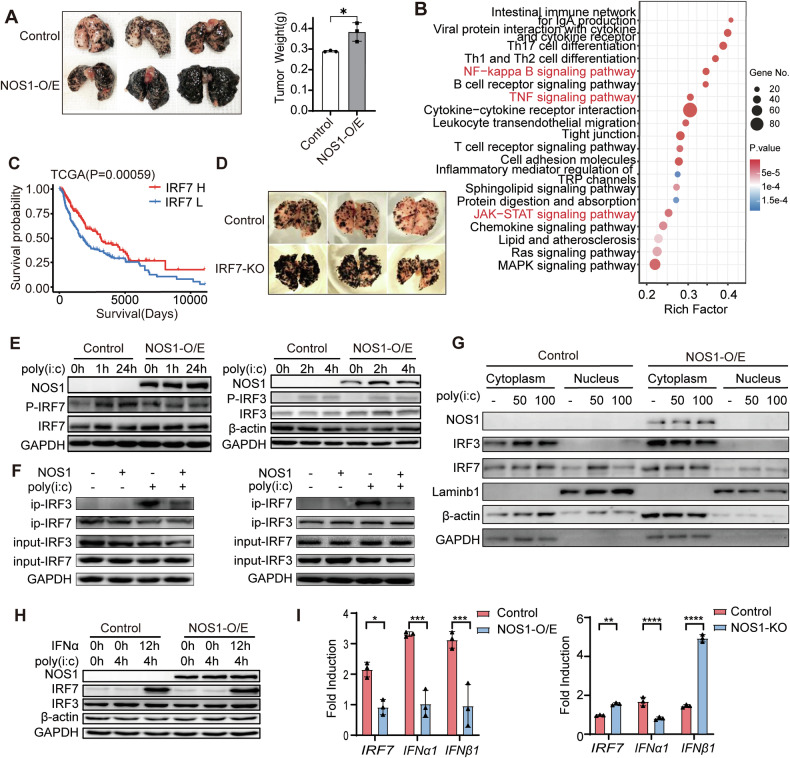
NOS1 expression inhibits Poly(I:C)-induced IFN-β transcription. **A** Gross morphology (left panel) and tumor weight (right panel) (*n* = 3) in the melanoma lung metastasis model showed the increased tumor growth in B16 NOS1-overexpression group compared to the control group. **B** KEGG pathway analysis of deferentially expressed genes (*p* < 0.01; fold change >1.2) between high and low NOS1 expression groups in melanoma patients from the TCGA database showed enrichment in cellular immune response signaling and IFN signaling (Student’s *t* test). **C** Kaplan–Meier analysis showed the increased overall survival in melanoma patients (TCGA cohort; *n* = 425) with high IRF7 expression. *p* values were determined using the log-rank test. **D** Gross morphology in the C57BL/6J mouse melanoma lung metastasis model showed the decreased tumor growth in B16 IRF7-KO group compared to the control group (*n* = 3). **E** Western blot analysis showed a slight decrease in phosphorylated IRF7 (left panel) in NOS1-O/E A375 cells, whereas the level of phosphorylated IRF3 (right panel) remained unchanged. **F** Co-IP assays revealed a decreased binding of IRF7 to IRF3 (left panel) and of IRF3 to IRF7 (right panel) in NOS1-overexpressing A375 cells. **G** Western blot analysis showed decreased expression of nuclear IRF7 proteins in NOS1-O/E A375 cells with 50 μg/mL Poly(I:C). Data are represented as mean ± SD. Data are representative of three independent experiments. **H** Western blot analysis showed no difference between Control and NOS1-O/E A375 cells in IRF7 and IRF3 level induced by treatment with IFNα and Poly(I:C). **I** qPCR analysis demonstrated that the fold induction of *IRF7* and *IFNβ* decreased in NOS1-overexpressing A375 cells (left panel) and increased in NOS1-knockout A375 cells (right panel) following treatment with Poly(I:C) compared to untreated cells (two-way ANOVA). ns, not significant *p < 0.05; **p < 0.01; ***p < 0.001; ****p < 0.0001.

We utilized genetically modified NOS1 melanoma cells (either overexpressing or knocked out NOS1 in the mouse B16F10 melanoma cell line) to establish a lung metastasis model. Compared to the control group, the NOS1-overexpressing B16F10 cells formed more and larger metastatic nodules in the mouse lungs (Fig. 1A), while the NOS1-knockout B16F10 metastatic tumors grew more slowly and formed fewer nodules. Our previous studies indicated that the transcriptional changes associated with NOS1 expression in melanoma were enriched in immune response pathways, particularly those related to IFN signaling [30]. Analysis of the TCGA melanoma database showed that NOS1 expression is positively associated with several immune-related pathways, including the JAK-STAT, NF−κB, and TNF signaling pathways, suggesting a potential link between NOS1 activity and immune modulation (Fig. 1B). These findings suggest that NOS1 may target the IFN signaling pathway to regulate immune responses in melanoma.

Interferon regulatory factor 7 (IRF7) is a transcription factor that plays a crucial role in the activation of type I interferon (IFN) signaling. We evaluated the clinical relevance of IRF7 expression in melanoma using data from the TCGA database. The mRNA level of *IRF7* is higher in melanoma compared to normal control tissues (Supplementary Fig. 1A). High IRF7 expression correlates with increased infiltration of immune cells within tumors (Supplementary Fig. 1B) and is associated with improved survival prognosis of patients (Fig. 1C). However, the incidence of IRF7 mutations is the highest in melanoma among 19 types of solid tumors (Supplementary Fig. 1C). In the mouse lung metastasis model, IRF7 silencing significantly increased the number of B16F10 metastasis nodules and enhanced tumor growth (Fig. 1D), suggesting that IRF7 contributes to immune activity in melanoma.

To investigate whether NOS1 targets IRF7 to modulate IFN responses, we analyzed the effect of NOS1 on phosphorylation of IRF7 under Poly(I:C) (synthetic double-stranded RNA analogues Polyinosinic acid) stimulation. The expression level of IRF7 was very low in resting melanoma cells, so we upregulated the IRF7 level by treating cells with IFN-α prior to the Poly(I:C) stimulation. We observed that overexpression of NOS1 led to a reduction in the phosphorylation of IRF7, while it did not have a measurable impact on the phosphorylation levels of IRF3 (Fig. 1E). The degree of NOS1 inhibition on IRF7 phosphorylation increased with prolonged Poly(I:C) stimulation. Treatment with the NO donor GSNO produced similar results, while treatment with the NOS1-specific inhibitor N-PLA or the NOS pan-inhibitor L-NAME increased IRF7 phosphorylation. In contrast, the NOS2-specific inhibitor 1400 W had no effect (Supplementary Fig. 1D), indicating a selective regulatory role of NOS1 in modulating IRF7 activation. Following phosphorylation, IRF7 forms a dimer and translocates to the nucleus to drive *IFNB1* (encoding IFN-β) transcription. Co-immunoprecipitation experiments showed that Poly(I:C)-induced dimerization of IRF7 and IRF3 was reduced in NOS1 overexpressing melanoma cells (Fig. 1F). The nuclear localization of IRF7 was also reduced in NOS1 overexpressing melanoma cells, which was partially reversed by high-dose Poly(I:C) treatment (Fig. 1G). These results suggest that NOS1 acts as a reversible negative regulator of IRF7 activity.

IRF7 activates the transcription of the *IFNB1* gene to initiate IFN signaling under PRR pathway activation. We evaluated the impact of NOS1 on IRF7-induced *IFN-*β transcription in response to Poly (I:C). NOS1 overexpression significantly inhibited *IFNB1* and *IRF7* transcription in A375 melanoma cells in response to Poly(I:C) stimulation, and this inhibition was reversed by NOS1-knockout (Fig. 1H). Similar results were obtained in human ovarian cancer SKOV3 and colon cancer SW480 cells (Supplementary Fig. 1F). The production of *IFN-*β subsequently activates JAK/STAT1 signaling to transcribe *IRF7*, creating a positive feedback loop in IFN signaling. In melanoma cells, IRF7 is strongly induced by IFNα. Notably, in melanoma cells with NOS1 overexpression, the level of IRF7 induced by IFNα is similar to that observed in control cells (Fig. 1I), which suggests that the JAK/STAT1 pathway remains unaffected by NOS1. Thus, NOS1 inhibition of IFNs signaling activation in tumor cells primarily occurs during the initial phase of cellular response to PRR signaling, rather than through disruption of components in the JAK/STAT1 pathway.

### NOS1 induced the S-nitrosylation of IRF7 at the C435 site in B16F10 cells

**Fig. 2:**
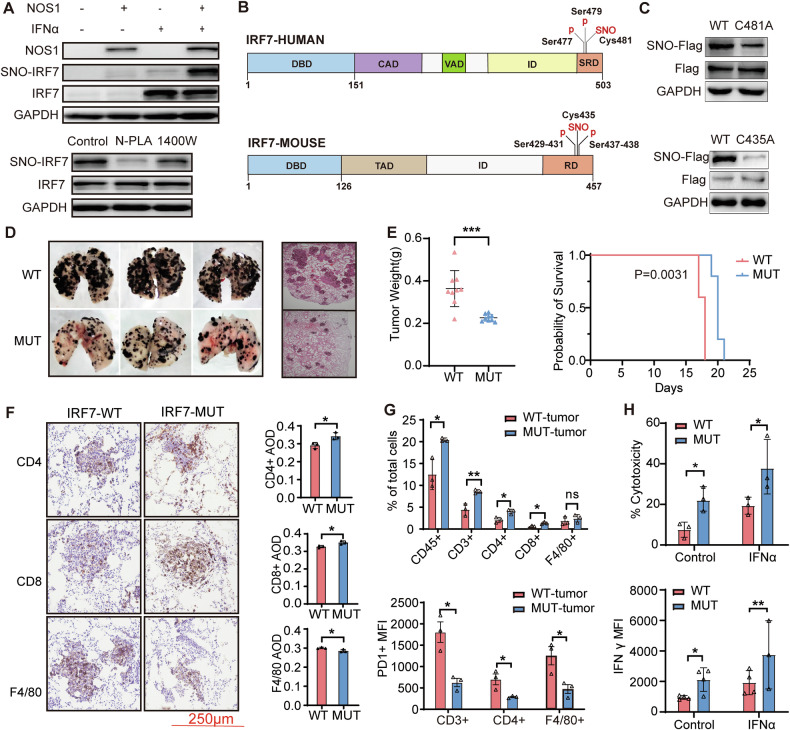
NOS1 induced the S-nitrosylation of IRF7 at the C435 site in B16F10 cells. **A** Biotin-switch assay showed the enhanced S-nitrosylation of IRF7 in NOS1-overexpressing A375 (upper panel) and decreased S-nitrosylation of IRF7 in NOS1-overexpressing A375 cells after IFNα (1000 U/mL) treatment 12 h (lower panel). **B** Diagrams of human IRF7 (upper panel) and mouse IRF7 (lower panel) domains and S-nitrosylation and phosphorylation sites. DBD DNA binding domain, CAD constitutive activation domain, VAD virus activated domain, ID inhibitory domain, SRD signal response domain, TAD transactivation domain, RD regulatory domain. **C** Biotin-switch assay showed decreased S-nitrosylation levels of IRF7 in A375 cells transfected with IRF7-C481A plasmid (upper panel), and B16F10 cells transfected with IRF7-C435A plasmid (lower panel). **D** Gross morphology (left panel) and H&E stained (right panel) showing the decreased tumor growth in lung IRF7-C435A-B16F10 metastasis (*n* = 3). **E** Decreased weight (left panel, *n* = 9) (Student’s *t* test)and increased survival (right panel, *n* = 5) (Log-rank test) of IRF7-C435A-B16F10 metastasis compare to the WT- B16F10 group. **F** Immunohistochemical staining (left panel) and average optical density (right panel) showed the increase of CD4- and CD8-T cells and decreased F4/80 macrophages in IRF7-C435A-B16F10 metastasis. Scale bar: 250 µm (Student’s *t* test). **G** Flow cytometry analysis showed the increased immune cell infiltration (upper panel) and mean fluorescence intensity of PD-1 (lower panel) in IRF7-C435A-B16F10 metastasis compared to the control group (Multiple *t*-test). **H** IFNγ expression detected by Flow cytometry (upper panel) and the cytotoxicity detected by CytoTox 96 Non-Radioactive Cytotoxicity Assay (lower panel) increased in splenocytes from IRF7-C435A-B16F10 bearing mouse after co-cultured with corresponding melanoma cells or stimulated with IFNα (two-way ANOVA). ns, not significant *p < 0.05; **p < 0.01; ***p < 0.001; ****p < 0.0001.

NOS1-derived NO can give rise to species such as N₂O₃ or NO⁺ to mediate S-nitrosylation of specific cysteine residues in target proteins, which is an essential process for its biological functions. We assessed the level of IRF7 S-nitrosylation and the impact of NOS1 expression on this modification in melanoma cells. A biotin-switch assay showed that IFN-α-induced IRF7 exhibited mild S-nitrosylation in A375 cells; however, overexpression of NOS1 resulted in a marked increase in the levels of IRF7 S-nitrosylation. This modification was only reduced by the NOS1-selective inhibitor (N-PLA), with no effect observed from the NOS2-selective inhibitor (1400 W) (Fig. 2A).

To identify the potential site of IRF7 S-nitrosylation, we used the GPS-SNO2.0 platform, which predicted human IRF7-C481 (highly homologous to mouse IRF7-C435) as the likely nitrosylation site (Fig. 2B). The evolutionarily conserved residue is situated near the phosphorylation site of IRF7. To validate that C481A in humans and C435 in mice serve as the primary targets for NOS1-mediated nitrosylation, we deleted endogenous IRF7 in melanoma cells and reintroduced alanine-substituted versions (human IRF7-C481A or mouse IRF7-C435A). In NOS1-overexpressing melanoma cells, we observed a decrease in the level of IRF7 S-nitrosylation in both human IRF7-C481A A375 cells and mouse IRF7-C435A B16F10 cells (Fig. 2C). This result indicates that this site is critical for the S-nitrosylation of target proteins and their associated biological functions.

RNA-seq assays were applied to evaluate the biological function of IRF7-C435 S-nitrification (IRF7-C435- SNO) in the regulation of cellular IFN signaling responses. We compared IRF7-C435A B16F10 cells with control B16F10 cells, both before and after IFNα stimulation. The expression levels of IFN-stimulated genes (ISGs) were significantly increased in IRF7-C435A groups (Supplementary Fig. 2A). These ISGs were enriched in immune response pathways, including the JAK/STAT pathway (Supplementary Fig. 2C, D). Moreover, when comparing with our previous RNA-seq data of NOS1 knockout B16F10, we noticed that IRF7-C435A cells shared similar characteristics with those in NOS1 knockout B16F10. These findings indicate that NOS1 targets IRF7-C435 to mediate dysfunction in IFN signaling in melanoma cells (Supplementary Fig. 2B).

We further investigated the immune regulatory role of IRF7-C435 S-nitrosylation in a melanoma mouse model. IRF7-C435A B16F10 cells had fewer and smaller nodules in lung metastasis, thus leading to improved survival prognosis compared to the control group (Fig. 2D, E). Tumor-infiltrating immune cells, including CD45^+^, CD3^+^, CD8^+^ and CD4^+^ T lymphocytes, were significantly increased in IRF7-C435A B16F10 tumors compared to the IRF7-WT groups, while the infiltration of F4/80 macrophages was decreased in the IRF7-C435A B16F10 tumors, as detected by flow cytometry (Fig. 2G) and immunohistochemical assays (Fig. 2F). In addition, we observed a significant reduction in the expression of PD-1 in CD3^+^, CD8^+^ and CD4^+^ T cells within the IRF7-C435A B16F10 tumors (Fig. 2G). Moreover, the levels of IFN-γ and granzyme B (GZMB) in spleen T lymphocytes, as well as their cytotoxic activity from IRF7-C435A B16F10 tumor-bearing mice, were significantly higher compared to those in the IRF7-WT group (Fig. 2H). However, no significant differences were observed in the proportions of immune cells in the spleen between the two groups (Supplementary Fig. 2F). These results suggest that NOS1-mediated IRF7-C435 S-nitrosylation impairs T cell infiltration and reduces cytotoxic activity in the tumor microenvironment.

### IRF7 S-nitrosylation down-regulates antigen presentation process in melanoma cells

**Fig. 3:**
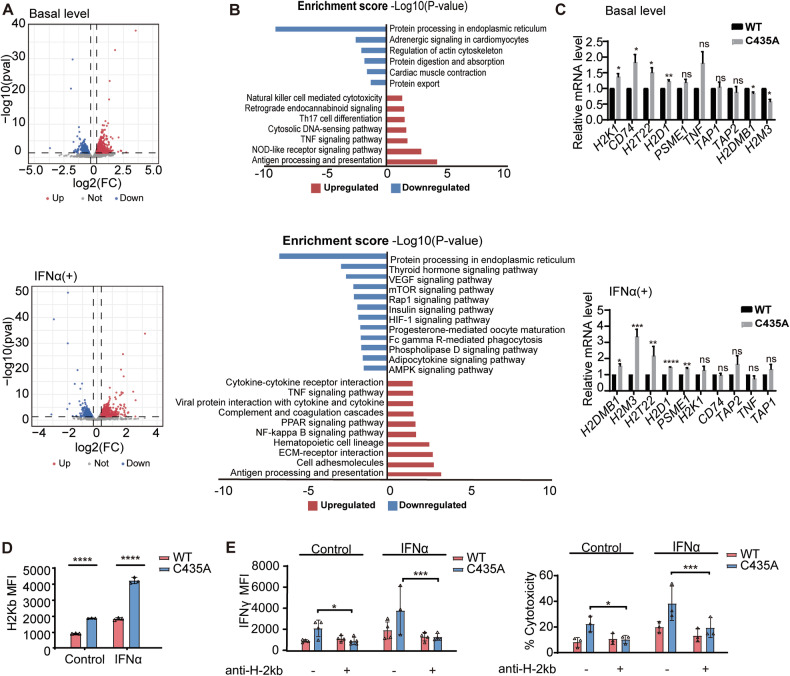
IRF7 S-nitrosylation down-regulates antigen presentation process in melanoma cells. **A** Volcano plot of the transcriptional data of IRF7-C435A vs. WT melanoma A375 (left panel) and KEGG analysis displays the upregulated pathway by compare IRF7-MUT vs. IRF7-WT (right panel). **B** Volcano plot of the transcriptional data of IRF7-C435A vs. WT melanoma A375 (left panel) and KEGG analysis displays the upregulated pathway by compare IRF7-MUT vs. IRF7-WT (right panel) under IFNα treatment. **C** The expression of 10 genes in Antigen processing and presentation signaling at basal levels (up panel) and under IFNα treatment (lower panel) by qPCR assays (Multiple *t*-test). **D** Flow cytometry analysis showed an increase of H2KB expression in IRF7-MUT cells compare to IRF7-WT with or without IFN alpha stimulation (two-way ANOVA). **E** The intensity of IFNγ detected by flow cytometre (left panel) and T cell cytotoxicity detected by CytoTox 96 Non-Radioactive Cytotoxicity Assay (right panel) in co-cultures system (B16-IRF7-WT or B16-IRF7-MUT tumor bearing splenocytes with corresponding tumor cells were decreased by treatment with H2KB antibody. (two-way ANOVA). ns, not significant *p < 0.05; **p < 0.01; ***p < 0.001; ****p < 0.0001.

We identified 1970 differential expressed genes (DEGs), between the transcription data of IRF7-C435A mutant and IRF7-WT A375 cells, including 1018 up-regulated and 1147 down-regulated genes. The up-regulated DEGs were enriched in immune response signaling pathways, while the down-regulated DEGs were enriched in metabolic processes. KEGG pathway analysis showed that the basal DEGs were involved in antigen presentation and processing (Fig. 3A). After IFN-α treatment, we identified 2165 DEGs between IRF7-C435A mutant and IRF7-WT A375 cells, comprised of 965 up-regulated and 1005 down-regulated genes. The up-regulated DEGs included genes associated with the regulation of MHC Class I and II antigens, as well as components involved in multiple antigen presentation processes (APM, Fig. 3B). The mutation in IRF7 also resulted in the up-regulation of immunoproteasome subunits Psmb9 and Psmb10, while Psmb8 remained unchanged. Additionally, there was a down-regulation of several components of the catalytic proteasomal core, including Psmd1, Psmd14, Psmc1, Psmc6, and Psmc2. Moreover, the IRF7 mutation led to the up-regulation of the subunits of the transporter associated with antigen processing (TAP1 and TAP2), suggesting an increase of the transport of peptides into the lumen of the endoplasmic reticulum (ER) for the assembly of the MHC-I-β-2-microglobulin (B2M) complex on the cell surface (Supplementary Fig. 3A).

To confirm the role of the IRF7-C435A mutation in upregulating APM, we examined the expression of MHC antigens and APM components in A375 cells. Real-time quantitative PCR showed that five out of ten genes were significantly up-regulated in IRF7-C435A-B16 cells with or without IFNα stimulation (Fig. 3C). Flow cytometry showed that the H2KB protein level was significantly higher in the B16-IRF7-C435A cells, both with and without IFNα stimulation (Fig. 3D). In addition, the transcriptome changes caused by the IRF7-C435A mutation were consistent with those observed upon NOS1 knockout and were focused on APM processes [30]. Therefore, the down-regulation of tumor cell antigen presentation appears to be the most important tumor biological function of IRF7-C435A S-nitrosylation induced by NOS1 expression in melanoma cells.

The presence of MHC-I molecules is essential for the immune recognition and activation of cytotoxic T lymphocytes (CTLs). Blocking antigen presentation with H2KB antibodies reduces the cytotoxic function of lymphocytes against the corresponding melanoma cells. In our co-culture system, both the cytotoxic function and mean fluorescence intensity (MFI) of IFN-γ-positive lymphocytes from IRF7-C435A B16F10 tumor-bearing mice were significantly enhanced compared to lymphocytes from IRF7-WT tumor-bearing mice. However, this enhanced cytotoxicity of IRF7-C435A lymphocytes was reduced by the addition of H2KB antibodies (Fig. 3E), suggesting that downregulation of antigen presentation is the main mechanism by which NOS1-mediated S-nitrosylation of IRF7 facilitates tumor escape from killing by immune cells.

### B16-IRF7MUT reverses immune suppression in the tumor micro-environment

**Fig. 4:**
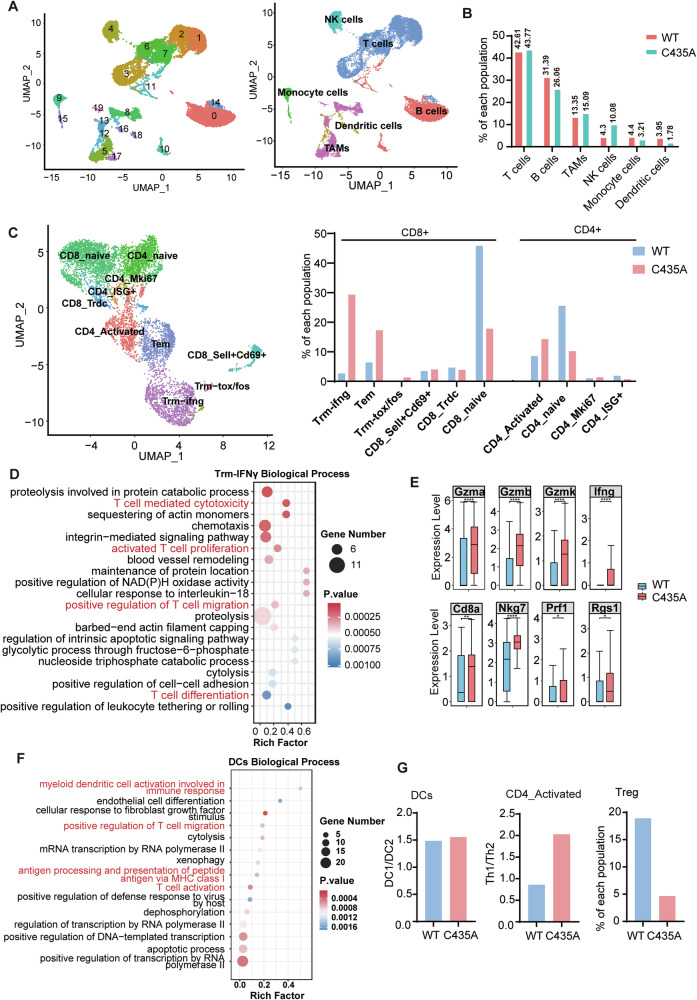
B16-IRF7MUT reverses immune suppression in the tumor micro-environment. **A** Clustering of intratumoral CD45+ cells and visualization of the subsets. Uniform manifold approximation and projection (UMAP) analysis of single-cell RNA sequencing data from 18,373 cells (WT: 9064; MUT: 9309) revealed 20 distinct clusters, labeled as cluster 0–19 (left panel) and cell type annotation plot six cell types determined by 20 specific markers (Supplementary Fig. 4). Each dot plot represents one cell (right panel). **B** The percentages of CD45+ subsets (T cells, B cells, TAMs, NK cells, monocytes and DCs) in the WT and C435A groups. **C** Subclustering of T lymphocytes and visualization of the subsets. Dimension reduction by UMAP displaying ten subsets of T lymphocytes (7937 cells) (left panel) and the percentages of T lymphocytes subsets in the WT and C435A groups (right panel). **D** Biological process analyses of 198 upregulated genes (fold change >1.2, *p* value < 0.05) in CD8-Trm-IFNγ T cells. The rich factor indicates the number of significant genes associated with the Gene Ontology (GO) term divided by the total number of genes in the related pathway in the database. **E** Violin plot revealed Cd8a, Gzma, Gzmb, Gzmk, Nkg7, IFNγ, Prf1 and Rgs1 expression levels in CD8-Trm-IFNγ T cells increased in C435A group. Each dot represents one cell (Kruskal test). ns, not significant *p < 0.05; **p < 0.01; ***p < 0.001; ****p < 0.0001. **F** Biological process analyses of 194 upregulated genes (fold change >1.2, *p* value < 0.05) in Dendritic cells. **G** The proportion of DC1/DC2 and TH1/TH2 cells in the WT and C435A groups (left panel). The percentages of Treg cells in CD4_Activated population.

Using single-cell sequencing, we evaluated the regulatory role of IRF7-SNO in the tumor microenvironment. We conducted a comparative analysis of immune cells from pulmonary tumor nodules formed by B16-IRF7C435A and B16-IRF7WT melanocytes. A total of 18,373 immune cells were obtained from B16-IRF7 C435A and B16-IRF7WT cell-derived lung metastatic tumors and were clustered into 20 clusters (0–19) (Fig. 4A). Based on the classical marker genes used in published literature and the annotated information in the PanglaoDB database, these 20 clusters were classified into six types of immune cells: T cells (cluster 1, 2, 3, 6, 7, 14), B cells (cluster 0, 10, 11), macrophages (cluster 5, 8, 13, 17, 19), Natural killer (NK) cells (cluster 4), monocytes (subgroup 9, 15), and dendritic cells (DCs) (cluster 12, 16, 18) (Fig. 4A and Supplementary Fig. 4A). Compared with the WT group, the proportion of NK cells in the C435A group was increased, while the proportion of DCs was decreased. The proportions of T cells, B cells, tumor-associated macrophages (TAMs), and monocytes were similar between the two groups (Fig. 4B).

Effector T cells residing in the tumor microenvironment are the main effector cells against tumors, and their expansion and function are critical for antitumor effects. All T cells are further classified into ten categories according to T cell markers, including activated CD8^+^ T cells (Tem, cluster 2; Trm-ifng, cluster 4, 6; Trm-tox/fos, cluster 11), activated CD4^+^ T cells (CD4_Activated, cluster 1), naive T cells (CD8_Naive, cluster 0, 5 and CD4_Naive, cluster 1), and other T cells with undefined functions (CD8_Trdc, cluster 7; CD8_Sell^+^Cd69^+^, cluster8; CD4_ISG^+^, cluster 9 and CD4_Mki67, cluster 1) (Supplementary Fig. 4B). Activated CD8^+^ T cells, especially the resident memory effect T cells, predominate in IRF7-C435A mutated tumor, while CD8_Naive and CD4_Naive predominate in IRF7-WT tumors (Fig. 4C). After the IRF7-C435 mutation, the proportion of CD8_activated cells increased significantly, from 9.14 to 47.88%. Among these, Trm-Ifnγ cells accounted for the majority. These cells are a subset of tissue-resident memory T cells that secrete IFN-γ and are crucial for local immune responses against infections and tumors. By producing IFN-γ, these cells can quickly activate immune mechanisms, serving as an essential component of the rapid immune response system in tissues. The Gene Ontology (GO) biological processes of Trm-Ifnγ cells show that IRF7-C435A-derived Trm-Ifnγ cells upregulated genes primarily involved in T cell-mediated cytotoxicity, activated T cell proliferation, and positive regulation of T cell migration (Fig. 4D). The expression levels of *Cd8a*, *Gzma*, *Gzmb*, *Gzmk*, *Ifng*, *Nkg7*, *Rgs1*, and *Prf1* in the IRF7-C435A group were significantly higher than those in the WT group (Fig. 4E). Therefore, NOS1-induced IRF7-SNO inhibits T cell activation and T cell killing in the tumor immune microenvironment.

B16-WT-derived dendritic cells (DCs) accounted for 3.95% of immune cells, whereas B16-C435A-derived DCs constituted only 1.78% (Fig. 4B). Mature DCs efficiently present antigens for the effectively initiation of T cell activation, while immature DCs induced T cells exhaustion, thereby creating an immunosuppressive tumor microenvironment. Out data showed that the up-regulated genes in B16-C435A-derived DCs were primarily enriched in antigen presentation processes of myeloid DCs during immune responses (Supplementary Fig. 4D), and the activation of myeloid DCs was revealed through biological process analysis (Fig. 4F). Although the ratio of DC1 to DC2 subgroups was not significantly affected by IRF7-C435A mutation, there was a notable increase in the ratio of T_H1_ to T_H2_ in the cluster of activated CD4^+^ T cells, accompanied by a decrease in T_reg_s (Fig. 4G and Supplementary Fig. 4E). These data suggest that B16-IRF7-C435A reversed the immune suppression in the tumor microenvironment.

### Clinical correlations exist between NOS1 expression and tumor immunity

**Fig. 5:**
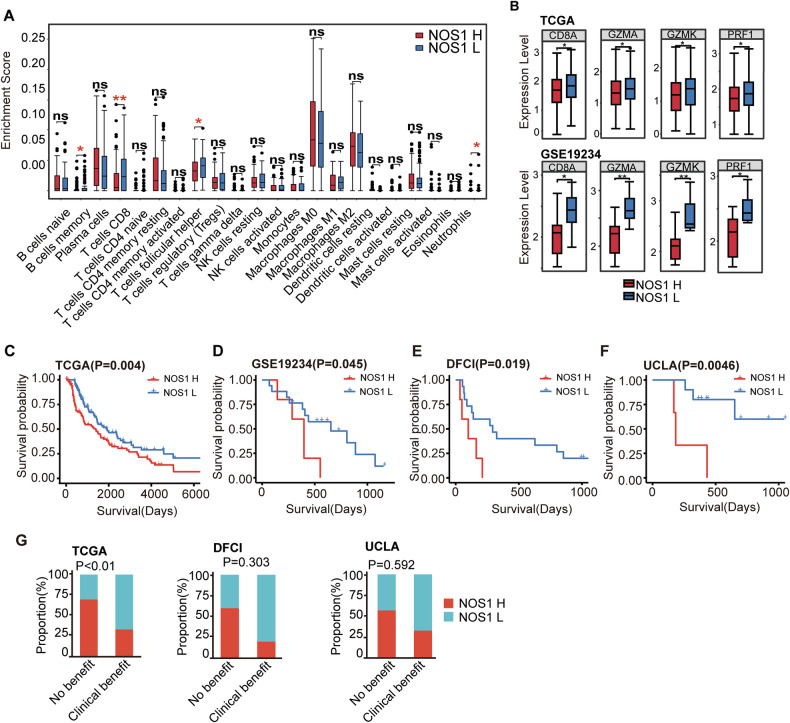
Clinical correlations exist between NOS1 expression and tumor immunity. **A** CIBERSORT analysis of differences in immune enrichment scores showed increased CD8 + T cell infiltration in the low expression group of NOS1 in melanoma patients from the TCGA database with low CD45 expression (median, *n* = 212) (Wilcox test). **B** Box plots showed increased expression levels of CD8A, GZMA, GZMK, and PRF1 in the low NOS1 expression groups in melanoma patients from the TCGA database (upper panel) and the GSE19234 database (lower panel) with low CD45 expression (Kruskal test). Kaplan–Meier analysis showed increased overall survival in melanoma patients with low CD45 expression and low NOS1 expression in both the TCGA cohort (**C**) and the GEO cohort (GSE19234 (**D**), DFCI (**E**), UCLA (**F**)) (Log-rank test). **G** The relationship between NOS1 expression and treatment outcomes in melanoma patients with low CD45 expression in the TCGA, DFCI, and UCLA databases showed a higher proportion of clinical benefit in patients with low NOS1 expression (Chi-square test). ns, not significant *p < 0.05; **p < 0.01; ***p < 0.001; ****p < 0.0001.

Using melanoma transcriptional profiles from public databases, we evaluated the effect of NOS1 expression on the composition and function of immune cells in the tumor micro-environment and its impact on immunotherapy outcomes in tumor patients. Considering that NOS1 is also expressed in tumor-infiltrating immune cells [46–50], we divided melanomas into high and low immune-infiltrating groups based on CD45 expression, stratified by the median CD45 expression level. In the TCGA data, the proportion of tumor-infiltrating CD8^+^ T cells was significantly reduced in the high NOS1 expression subgroup only in the melanoma group with low CD45 expression, but not in the CD45 high melanoma group (Fig. 5A). The expressions of *CD8A*, *GZMA*, *GZMK* and *PRF1* genes in the NOS1-high-expression group were significantly lower than those in the NOS1-low-expression group (Fig. 5B), indicating that NOS1 might play an immunoregulatory role only in “cold” melanoma. We analyzed the association of NOS1 transcription with survival outcomes in the TCGA and GEO databases. In samples with low CD45 expression, NOS1 expression was associated with poor survival outcomes in the TCGA database (Fig. 5C), but not in the high CD45 group (Supplementary Fig. 5A). Other transcription data from multiple GEO databases yielded similar results (Fig. 5D–F).

The downregulation of the major IFN and MHC Class I antigen-processing machinery components could result in a lack of response to IFN and immune checkpoint inhibitor therapy. In TCGA samples, we found that NOS1 expression was correlated with various treatment outcomes after excluded high CD45 expression samples. Patients with high NOS1 expression accounted for 69.4% of those who did not benefit clinically, significantly higher than the 27.7% observed in patients who benefited clinically. Similar results were observed in DFCI samples of melanoma patients receiving anti-CTLA-4 therapy and UCLA samples of patients receiving anti-PD-1 therapy (Fig. 5G). These data indicate that NOS1 negatively regulates the IFN gene signaling pathway, correlating with poor prognosis in immunotherapy.

## Discussion

The role of IRF7 in somatic cells is essential for IFN responsiveness. IRF7 is expressed at trace levels in somatic cells and is activated through the PRR signaling pathway in response to pathogens or cellular stress, resulting in the transcription of IFNβ [11]. After IFNβ is secreted outside the cell, it binds to the IFN receptor through a paracrine or autocrine mechanism, initiating the IFN signaling pathway, and further transcribing a large amount of IRF7, thus forming a positive feedback regulation mechanism of IFN signaling. In this study, we found that a small amount of IRF7 in resting tumor cells can be modified by NOS1-mediated S-nitrosylation, thereby inhibiting IRF7-mediated transcription of *IFNB1*, resulting in impaired activation of the type I IFN signaling in melanoma cells. Our study showed that stable expression of NOS1 in melanoma cells induced dysfunction of IRF7 through S-nitrosylation, thereby completely impairing the type I IFN response of melanoma cells. Clinically, NOS1 expression significantly reduced the infiltration of CD8^+^ T cells in the tumor microenvironment and was negatively correlated with the survival prognosis of cold melanoma patients. Therefore, NOS1 expression contributes to melanoma development by facilitating immune evasion.

Although NOS2 and NOS3 can produce NO, our results suggest that they do not significantly contribute to IRF7 S-nitrosylation in this system. Only the manipulation of NOS1, and not of NOS2/3, affected IRF7 modification, suggesting that the NOS1 subtype may function uniquely and effectively. The structural difference between NOS1 and the other two NOS isoforms lies in the presence of a PDZ domain at the N-terminal. NOS1 uses the PDZ domain to interact with other PDZ-containing proteins. This specific binding allows NOS1 to easily associate with target proteins, either directly or indirectly, selectively inducing S-nitrosylation of those targets. Our experiments indicated that the inhibition of IFN signaling by NOS1 is unique, because only NOS1-specific inhibitors, not those for NOS2 or NOS3, significantly affected the level of IRF7 S-nitrosylation. Although we did not detect direct binding of NOS1 to IRF7, it remains uncertain whether NOS1 binds to IRF7 indirectly via hinge proteins with PDZ domains(It remains to be determined whether NOS1 binds IRF7 indirectly through hinge proteins with PDZ domains.). Thus, NOS1 expression may endow melanoma with cold tumor characteristics, leading to IFN treatment and resistance to immunotherapy.

Decreased or absent expression of MHC class I molecules has been observed with a wide range of solid and hematopoietic tumors, leading to escape from T cell-mediated immune surveillance. In the context of the immune response, tumor cells respond to cellular IFN-I via autocrine or paracrine mechanisms. The activation of adaptive immune responses by IFNs is mainly mediated through the transcriptional activation of MHC class I and class II antigens and antigen-processing machinery components. In this study, IRF7-SNO disrupts MHC-I peptide presentation and increases protein degradation via ubiquitin-proteasome, leading to evasion of recognition and killing by cytotoxic T lymphocytes (CTLs). Moreover, our single-cell sequencing detected a reversal of tumor immune inhibitory environments. Disruption of IRF7-SNO through the C435 mutation promotes the maturation of DCs and increased the TH1/TH2 ratio, accompanied by enhanced abundance and improved function of CD8^+^ T cells at tumor sites, as well as augmented cytotoxic activity of NK cells. This suggests that NOS1 induction of IRF7 S-nitrosylation activates antigen-presenting cells (APCs) by increasing the transcription of MHC class I and class II antigens and APM components, thereby reversing immune suppression in the tumor microenvironment. Moreover, IFN-I in the tumor microenvironment acts as a “third signal” to activate initial CD8^+^ T cells. In the absence of this third signal, CD8^+^ T cells exhibit poor viability, resulting in limited clonal expansion. Our findings suggest that pharmacological inhibition of NOS1, or interventions that prevent IRF7 nitrosylation, could mimic the effect of the C435A mutation and thereby enhance antitumor immunity. This provides a rationale for considering NOS1 inhibitors as potential adjutant strategies which could enhance the efficacy of immune checkpoint blockade therapies. This possibility is being explored in ongoing studies.

Endogenous NO induces modifications in target proteins involved in cancer progression through various biological functions. Each nitric oxide synthase (NOS) has its own specific role due to different mechanisms of intracellular localization and activity regulation. NOS1 is constitutionally expressed and produces low levels of NO, which can induce S-nitrosylation of cysteine residues on target proteins. In contrast, NOS2, which is expressed in somatic and immune cells, can produce high concentrations of NO in response to inflammatory stimuli and damaging factors. High levels of NO can be toxic to cells or can react with superoxide ions in the environment to induce nitration modification of tyrosine residues on target proteins. In the context of melanoma, studies indicate that NOS2 promotes tumor proliferation and is associated with poor patient survival and increased resistance to cisplatin [51–54]. NOS3 uncoupling has been identified as a key mediator of melanocyte malignant transformation associated with sustained stress conditions, driving malignant transformation [55, 56]. We previously reported that NOS1 inhibits the expression of interferon-stimulated genes by nitrosating histone deacetylase 2 (HDAC2). Here, we showed that NOS1 induces tumor immune escape through S-nitrosylation of IRF7. NOS1 has multiple targets in melanoma and the NO produced by the three NOS isoforms may have overlapping effects. However, the specific roles of different NOS isoforms in melanoma development and their synergistic effects remain to be studied. Understanding the sources of NO and its spatiotemporal profile within the tumor microenvironment could enhance the efficacy of cancer immunotherapies.

## Materials and methods

### Cell culture

Human melanoma A375 cell line, murine melanoma B16F10 cell line, human colorectal cancer cell lines SW480, and human ovarian cancer cell lines SKOV3 were maintained in our laboratory. The cell lines were authenticated by STR profiling and regularly tested for mycoplasma contamination. Only mycoplasma-free cells were used for experiments. Cells were cultured in DMEM or RPMI-1640 medium (Gibco, Gaithersburg, MD, USA) supplemented with 10% fetal bovine serum (BI, Salt Lake City, UT, USA), and maintained at 37 °C in a humidified atmosphere of 5% CO_2_.

Stable NOS1 overexpression and nontargeted control cell lines were generated according to a previously reported method [57]. EX-I1936-Lv216–IRF7 (WT, C481A-MUT, Flag-tagged, human) and pLVX-mCherry-C1-IRF7 (WT, C435A-MUT, Flag-tagged, mouse) were designed and synthesized from IGEbio Technologies (Guangzhou, China).

The chemicals GSNO, N-PLA, L-NAME, and 1400 W were obtained from Cayman Chemical (Ann Arbor, MI, USA). Human and mouse IFNα (Sigma-Aldrich, St. Louis, MO, USA) was used to treat the cells for the indicated duration of time at a concentration of 1000 units per mL.

### RNA extraction and quantitative PCR (qPCR)

Total RNA was extracted from cultured cells or tumor tissue, and cDNA was synthesized using RNAiso Plus reagent (Takara, Shiga, Japan) and PrimeScript RT kit (Takara), respectively. qPCR was performed on a LightCycler 96 System (Roche Life Science) using TB Green Premix Ex Taq II (Takara). All samples were normalized to the endogenous control GAPDH, and relative fold expression levels were calculated using the 2^−ΔΔCt^ method. All experiments were performed independently at least three times, with all samples being analyzed in triplicate. The specific primers for RT-qPCR are as follows:

*IFNα4* forward, 5’ -GACCTGCCTCACACTTAT-3’, *IFNα4* reverse, 5’-AATCCTTCCTGTCCTTCA-3’, *IFNβ1* forward, 5’ -GTTCCTGCTGTGCTTCTC-3’, *IFNβ1* reverse, 5’-ATCTTCTCCGTCATCTCCAT-3’, *IRF7* forward, 5’ -TGAGCGAAGAGAGCGAAGAG-3’, *IRF7* reverse, 5’-CCAGTAGATCCAAGCTCCCG-3’, *GAPDH* forward, 5’ -CTACCCCCAATGTGTCCGTC-3’, *GAPDH* reverse, 5’-TGAAGTCGCAGGAGACAACC-3’, *H2-K1* forward, 5’ -GGCAATGAGCAGAGTTTCCGAG-3’, *H2-K1* reverse, 5’-CCACTTCACAGCCAGAGATCAC-3’, *Cd74* forward, 5’ -CGAGAGCTGGATGAAGCAGT-3’, *Cd74* reverse, 5’-GTTACCGTTCTCGTCGCACT-3’, *H2-DMb1* forward, 5’ -GCTGTATCCATGGGCCGAAAAT-3’, *H2-DMb1* reverse, 5’-AAGGTGTGTGGTTTGGGCTACT-3’, *Tap2* forward, 5’ -CTGGCGGACATGGCTTTACTT-3’, *Tap2* reverse, 5’-CTCCCACTTTTAGCAGTCCCC-3’, *Tnf*, forward, 5’-GGTGCCTATGTCTCAGCCTCTT-3’, *Tnf*, reverse, 5’-GCCATAGAACTGATGAGAGGGAG-3’, *H2-M3* forward, 5’ -CTCCGCTACTACAACCAGAGTG-3’, *H2-M3* reverse, 5’-TCTGAGCCATCATACGCAGCCT-3’, *H2-T10*, forward, 5’ -GGCTCGCAGACCCATGAAG-3’, *H2-T10* reverse, 5’-GGCCTAGACAAGTTTTACAGGAC-3’, *H2-T22* forward, 5’ -TCCCTTTGGGTTCACACTCG-3’, *H2-T22* reverse, 5’-AGTCGTCCATGCTCTTGTTGT-3’, *H2-D1*, forward, 5’ -TGAGGAACCTGCTCGGCTACTA-3’, *H2-D1* reverse, 5’-GGTCTTCGTTCAGGGCGATGTA-3’, *Psme1* forward, 5’ -TCACTACCTGGTTGCAGCTACAG-3’, *Psme1* reverse, 5’-GGTGTGAAGGTTGGTCATCAGC-3’, *Tap1* forward, 5’ -GGACTTGCCTTGTTCCGAGAG-3’, *Tap1* reverse, 5’-GCTGCCACATAACTGATAGCGA-3’, *Psme2* forward, 5’ -TCCTCTGCACTTTCTTGCCACG-3’, *Psme2* reverse, 5’-TGGGATAGGGATGTCCAGAGGA -3’.

### Western blotting

Total protein was extracted using a Protein Extraction Kit (Fude, Hangzhou, China). Protein concentration in each sample was determined using the Bradford assay (Beyotime, Shanghai, China). Equal amounts of protein were separated on 8–15% SDS-PAGE gels and then transferred to PVDF membranes (Millipore, USA). The membranes were blocked with 5% BSA for 1 h. After blocking, the membranes were incubated with primary antibodies: anti-NOS1 (Abcam, Cambridge, MA, USA) at a 1:1000 dilution and anti-GAPDH (Proteintech, Wuhan, China) at a 1:5000 dilution. Following incubation with HRP-conjugated secondary antibodies, the immunoreactive bands were visualized using chemiluminescence (Bio-Rad, USA).

### Coimmunoprecipitation (Co-IP)

Immunoprecipitates were obtained using the Co-Immunoprecipitation Kit (Cat. No.26149, Thermo Fisher), as described previously [57]. The assay was performed according to standard procedures.

### S-nitrosylation detection assay

S-nitrosylated protein detection assays were performed as described previously [58]. Briefly, 100–250 μg protein lysates were extracted from A375 and B16 cells treated with IFNα (1000 U/mL), GSNO (100 μM), LNAME (1 mM), N-PLA (100 μM), or 1400 W(100 μM). Biotinylated proteins can be easily detected by biotin western blot or streptavidin precipitation, followed by western blotting.

### CRISPR-Cas9-mediated genome editing, lentivirus production and cell line selection

IRF7-KO cells were obtained using the CRISPR-Cas9 system as described previously [Genome engineering using the CRISPR-Cas9 system]. The guide RNA (gRNA1: aaccatagaggcacccaag, gRNA2: acaagtgttggtaacaggt) was cloned into the Cas9 vector (NEWMOL, Synbio Technologies). Guide RNA-encoding plasmids were transfected into B16F10 cells for 48 h as described above.

Transfected cells were selected with G418 (300 ng/mL) to generate stable clonal lines from single cells, and individual clones were picked and cultured. Gene defects were identified by RT-PCR and immunoblotting. Lentiviral production was performed based on a previously described protocol [Production and purification of lentiviral vectors]. Stable IRF7-WT/MUT expression cell lines were generated by IRF7-KO cells infected with a lentivirus vector encoding IRF7-WT/MUT. Stable clones were selected with puromycin (1.5 μg/mL).

### Tumor models

C57BL/6 mice and BALB/c-nu mice (Female, 4–5 weeks old) were all purchased from Guangdong Medical Laboratory Animal Center. To construct a lung metastasis model of melanoma, 5 × 10^5^ B16F10 cells were intravenously injected into mice. After cell injection, the mice were randomly assigned to the experimental and control groups (5-8 mice per group), and they were then housed in SPF facilities on a 12 h light/ dark cycle until the end of the experiment. Mice were euthanized during days 15–17 postinjection, and lung tissue was isolated, photographed and then fixed with 4% formaldehyde for histological and morphometric measurements. In some cases, mice were sacrificed individually upon signs of metastatic distress and lung metastasis confirmed via histology and lung weight. The number of visible tumors in the lungs was counted separately, and fixed murine lungs were routinely processed and embedded in paraffin. Paraffin sections (5μm) were stained with H&E according to standard protocols, examined by microscopy and photographed.

### Flow cytometry

For analysis of tumor-infiltrating lymphocytes, resected tumor tissues were cut into small pieces and then digested in collagenase Ⅱ (1 mg/ mL) and collagenase Ⅳ (2 mg/ mL) at 37 °C for 30 min. The mixture was filtered through a 70-μm strainer to prepare a single cell suspension. Cells were then washed twice with PBS and resuspended in PBS, and 1 × 106 cells were incubated with 3 μl antibody for 30 min at 4 °C in darkness. Wash the cells twice and perform the analysis on the FACS Calibur (BD Biosciences, USA). The anti-mouse CD3-PE-Cy7, CD8-FITC, CD45-APC-Cy7, F4/80-BV510, CD11b-BV650, IFN-γ-BV421, H2Kb-Alexa Fluor 647, CD279-PE, CD279- Granzymeb antibodies were all purchased from BD Biosciences. Data are represented as the percentage of lymphocytes as indicated.

### Immunohistochemistry

Tumor tissues from B16-IRF7-WT/MUT tumor-bearing mice were collected in optimal cutting temperature (OCT) compound. Tumor tissues were sliced at a thickness of 4 μm. The tissue was fixed with 3.7% paraformaldehyde, and endogenous peroxidase was inactivated with 3% H2O2 at room temperature. Angiogenesis was detected with anti-CD4(1:1000 dilution, Abcam), anti-CD8(1:2000 dilution, Abcam) and anti-F4/80(1:100 dilution, Abcam) antibody. The level of angiogenesis was assessed at a magnification of ×200. Five randomly chosen fields per sample from each mouse were evaluated.

### Gene expression profiling by RNA-seq

Total RNA was extracted using Trizol reagent (Thermo Fisher, 15596018) and purified using Dynabeads Oligo (dT) (Thermo Fisher) with two rounds of purification. A Poly(A) RNA sequencing library was prepared following Illumina’s TruSeq-stranded mRNA sample preparation protocol. RNA integrity was checked with Agilent Technologies 2100 Bioanalyzer. The mRNA was fragmented into short fragments using divalent cations under elevated temperature (94 °C) (NEB, cat. e6150). Then the cleaved RNA fragments were reverse-transcribed to cDNA by SuperScript™ II Reverse Transcriptase (Invitrogen, cat. 1896649). Paired-ended sequencing was performed on Illumina’s NovaSeq 6000 sequencing system. The reads containing sequencing adapters, sequencing primers, and sequences with q quality score lower than 20 were removed prior to assembly. The cleaned sequencing reads were aligned to the reference genome using the HISAT2 package, which built a database of potential splice junctions. Multiple alignments with a maximum of two mismatches were allowed for each read sequence. String Tie was used for assembling the aligned reads of individual samples. Transcriptomes from all samples were then merged to reconstruct a comprehensive transcriptome using a proprietary Perl script of LC Sciences (Houston, Texas, U.S.A.). FPKM reads and differential expressed genes were evaluated by StringTie and edgeR, respectively. The differentially expressed mRNAs and genes were selected with log2 (fold change) ≥0.26 or log2 (fold change) ≤−0.26, and with *p* values < 0.05.

### Single-cell RNA sequencing (scRNA-seq) by Chromium 10x Genomics

Tumor tissues from B16-IRF7-WT/MUT tumor-bearing mice were cut into 2 mm pieces, and tumor tissues were prepared into a single-cell suspension with collagenase Ⅱ (1 mg/ mL) and collagenase Ⅳ (2 mg/ mL) at 37 °C for 30 min. Next, red blood cells were removed with red blood cell lysis buffer, and the single-cell suspensions were resuspended in flow staining buffer. Finally, the cells were treated with an Fc blocker to reduce the nonspecific binding of the Fc receptor and antibodies. The cells were labeled with rat anti-mouse BV510CD45 and 7-AAD (Cat. No: 51-65875X; BD Pharmingen). A total of 5 × 105 live immune cells (CD45 positive and 7-AAD negative) were sorted and resuspended in sample buffer for single-cell capture. Live CD45+ immune cells were captured using a BD Rhapsody^TM^ system and lysed.

The overall cell viability was confirmed to be above 90%. Single cell suspensions were counted using a hemocytometer/Countess II Automated Cell Counter and concentration adjusted to 700–1200 cells/mL. Single-cell suspensions were loaded to 10x Chromium to capture 8000 single cells according to the manufacturer’s instructions of 10x Genomics Chromium Single-Cell kit (V3). The following cDNA amplification and library construction steps were performed according to the standard protocol. Libraries were sequenced on an Illumina NovaSeq 6000 sequencing system (paired-end multiplexing run, 150 bp) by LC-Bio Technology (Hangzhou, China) at a minimum depth of 20,000 reads per cell.

### Bioinformatics and computational biology analyses

Sequencing data were de-multiplexed and converted to FASTQ format using Illumina bcl2fastq software (2.20). Sample demultiplexing, barcode processing and single-cell gene counting were initially processed using CellRanger (7.1) and scRNA-seq data were aligned to Ensemble genome GRCm38 reference genome, a total of 20529 single cell captured from 3 B16-IRF7WT mice and 3 B16-IRF7MUT mice were processed using 10X Genomics Chromium Single Cell Solution. The Cell Ranger output was loaded into Seurat (3.1.1) for dimensional reduction, clustering, and analysis of scRNA-seq data. Overall, 18,373 cells passed the quality control threshold: all genes expressed in less than 1 cells were removed, number of genes expressed per cell >500 as low and <Inf as high cut-off, UMI counts less than Inf, the percent of mitochondrial-DNA derived gene-expression <25%. To visualize the data, the dimensionality of all 18,373 cells was reduced using Seurat and t-SNE was used to project the cells into 2D space. Marker genes for each cluster were identified with the default parameters “bimod”: likelihood-ratio test with default parameters via the Find All Markers function in Seurat. This selects marker genes which are expressed in more than 10% of the cells in a cluster and average log(fold change) of greater than 0.26.

Bioinformatic analysis was performed using the OmicStudio tools at https://www.omicstudio.cn/tool. DEGs were screened by R statistical package software EdgeR (Empirical Analysis of Digital Gene Expression in R, http://www.bioconductor.org/packages/2.12/bioc/html/edgeR.html) with Fold Change >1.2 or < −1.2, FDR < 0.05. Kyoto Encyclopaedia of Genes and Genomes pathway enrichment analyses of DEGs were performed by the KOBAS online database (http://kobas.cbi.pku.edu.cn/kobas3).

Additionally, gene set enrichment analysis (GSEA) was performed using the “fgsea” package in R. We selected two GSEA databases for our analysis: hallmark gene sets and KEGG gene sets. Moreover, MetaCore software was used for pathway analysis of significant genes. The cluster annotations were established based on signature genes that were approved by previous studies. Highly expressed genes in CD8 + T cells were assessed using *f* tests, *t* tests, and *p* values < 0.05. A list of differentially expressed genes was imported into the Metacore software to construct signaling pathways. The significance cut-off point for enrichment of a pathway was a *p* value < 0.05.

### Statistical analysis

GraphPad Prism Software (version 9.0) was used for statistical analysis and preparation of most figures. Flow cytometry data analysis was carried out using FlowJo (version 10.0). ImageJ (version 2.3.0) was used for quantitative comparison of positive signals in Immunohistochemistry staining images. The choice of tests was based on the distribution and variance of the data. Normality was assessed using the Shapiro–Wilk test, and homogeneity of variances was tested using Levene’s test. For normally distributed data with equal variances, comparisons between two groups were made using the unpaired two-tailed Student’s *t* test. For non-normally distributed data, non-parametric tests (e.g., Mann–Whitney *U* test) were used. Two-way ANOVA with Tukey’s multiple comparison test was performed for multiple group comparison. The survival curves were performed by the Kaplan–Meier method and compared by the log-rank (Mantel–Cox) test, as indicated in the figure legends. The results are presented as mean ± SD unless otherwise noted. *p* < 0.05 was considered statistically significant. Mice were assigned to groups randomly. Analyses were not blinded, and no datapoints were removed from the analyses.

## Supplementary information

SUPPLEMENTAL figure

western blots

## Data Availability

Bulk RNA-Seq data are available data sets from NCBI’s Gene Expression Omnibus (GEO) at GSE279140. scRNA-Seq data are available data sets from NCBI’s Gene Expression Omnibus (GEO) at GSE279235. This study analyzed the existing bulk RNA-Seq data sets from TCGA, DFCI, UCLA and GEO at GSE19234, respectively (https://www.cbioportal.org/) [59–61]. Values for all data points in graphs are reported in the Supporting Data Values file.
