# Supplementary material for: S-nitrosylation of IRF7 induced by NOS1 expression in melanoma suppresses anti-tumor immunity
Source: Cell Death Dis. 2026 Jan 14;17(1):33. doi: 10.1038/s41419-025-08201-y (PMC12804790; doi:10.1038/s41419-025-08201-y)
Supplement: Supplementary file 1 — SUPPLEMENTAL figure [file 41419_2025_8201_MOESM1_ESM.pdf]

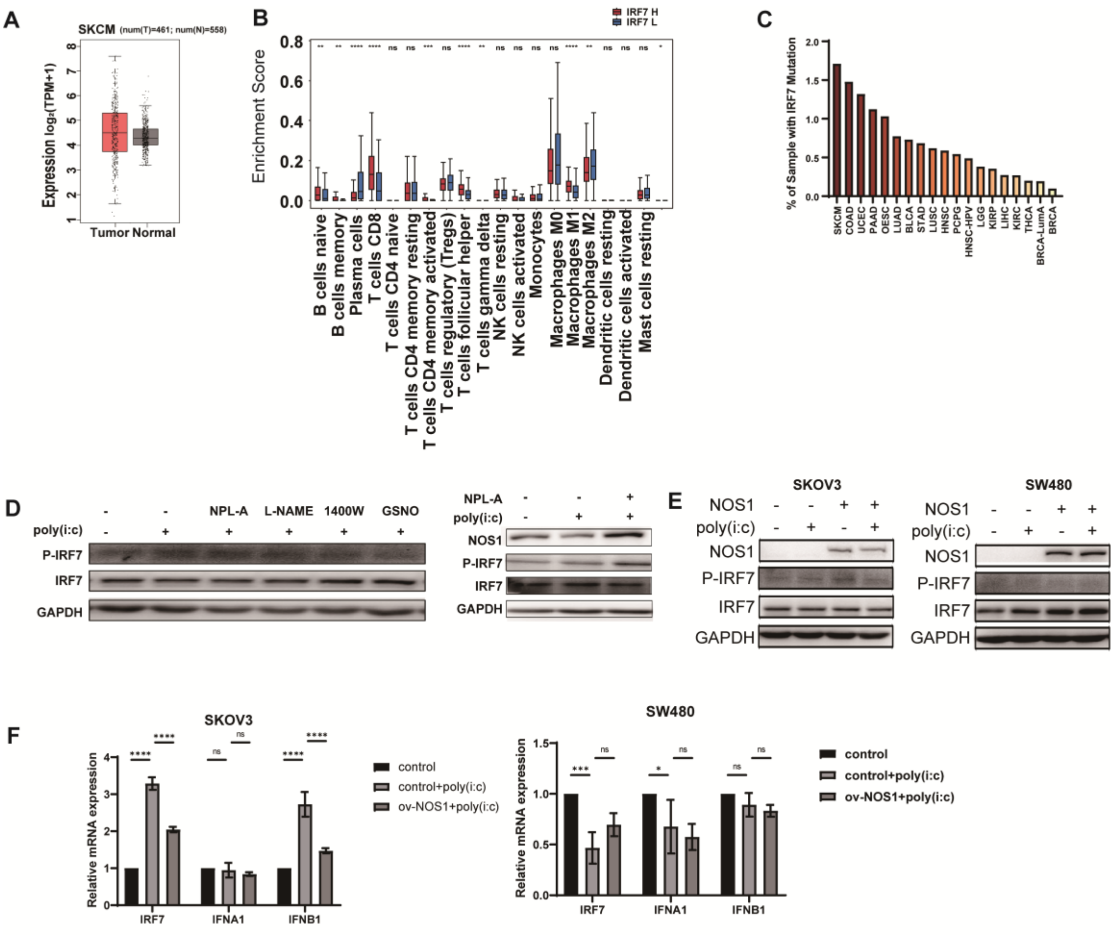

Supplementary Figure 1. NOS1 expression inhibits Poly(I:C)-induced IFN-β transcription

(A) Expression of IRF7 in melanoma from the TCGA database and normal tissues from the GTEX database.

(B) CIBERSORT analysis of immune enrichment scores showed the increased CD8+ T cell infiltration in the high IRF7 melanoma from the TCGA database .

(C) Mutation rates of IRF7 across various tumor samples in the TCGA database.

(D) qPCR analysis demonstrated that the induction of IRF7 and IFNβ in NOS1-overexpressing SKOV3 cells (left panel) and SW480 cells (right panel) .

(E) Western blot analysis showed a slight decrease in phosphorylated IRF7 in NOS1-O/E SKOV3 cells (left panel) and NOS1-O/E SW480 cells (right panel) remained unchanged. ns, not significant *P < 0.05; **P < 0.01; ***P < 0.001; ****P < 0.0001.

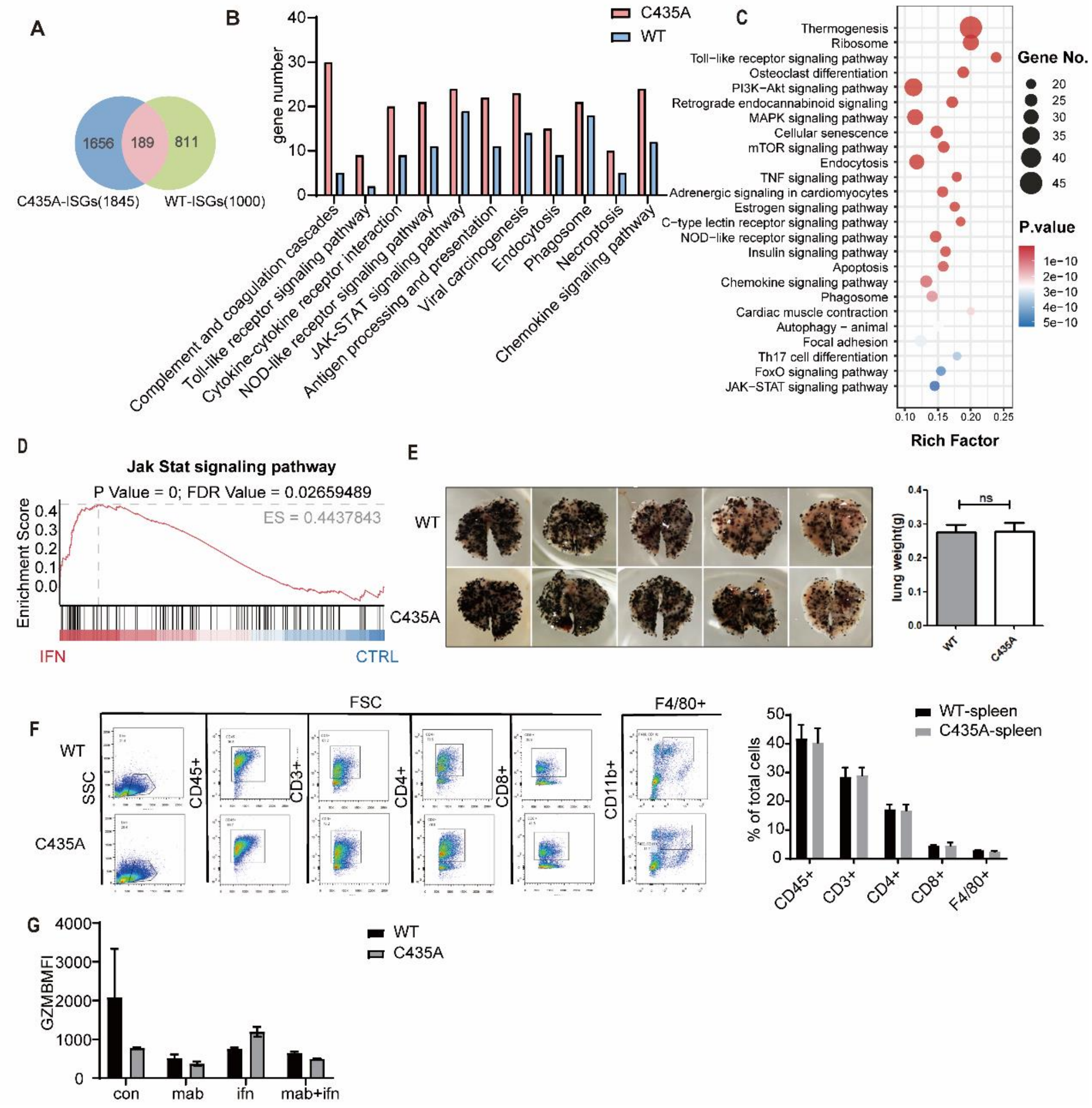

Supplemental Figure 2. NOS1 induced the S-nitrosylation of IRF7 at the C435 site in B16F10 cells

(A) The graph of IRF7-C435A group (IFNα- IRF7-C435A vs. IRF7-C435A) and WT group (IFNα- WT vs. WT) showed 1845 and 1000 IFN related genes.

(B) KEGG analysis showed that the gene number and pathways enriched in interferon stimulating genes (ISGs) were increased by IRF7-C435A (Red / Blue for IRF7-C435A / WT group).

(C) KEGG analysis revealed the top 25 most significantly enriched pathways of 1845 IFN-related genes in the IRF7-C435A group, including the Toll-like receptor signaling pathway, NOD-like receptor signaling pathway, and JAK-STAT signaling pathway.

(D) GSEA showed JAK-STAT signaling pathway up-regulated by IRF7-C435A

(E) Gross morphology (left panel) and tumor weight (right panel) in the melanoma lung metastasis model(n =5) showed no significant difference in tumor growth in IRF7-MUT

B16 tumor mouse compared to the control group.

(F) The subsets of spleen lymphocytes detected by flow cytometry showed that CD45, CD3, CD8, F4/80CD11b subsets enhanced in IRF7-C435A mutation groups.

(G) PD-1 expression on CD3, CD8, CD4 subsets enhanced in IRF7-C435A mutation groups detect by flow cytometry analysis. Representative flow cytometry plots (left panel), and quantification of PD-1 expression from 3 animals in each group (right panel). ns, not significant; * $P < 0.05$

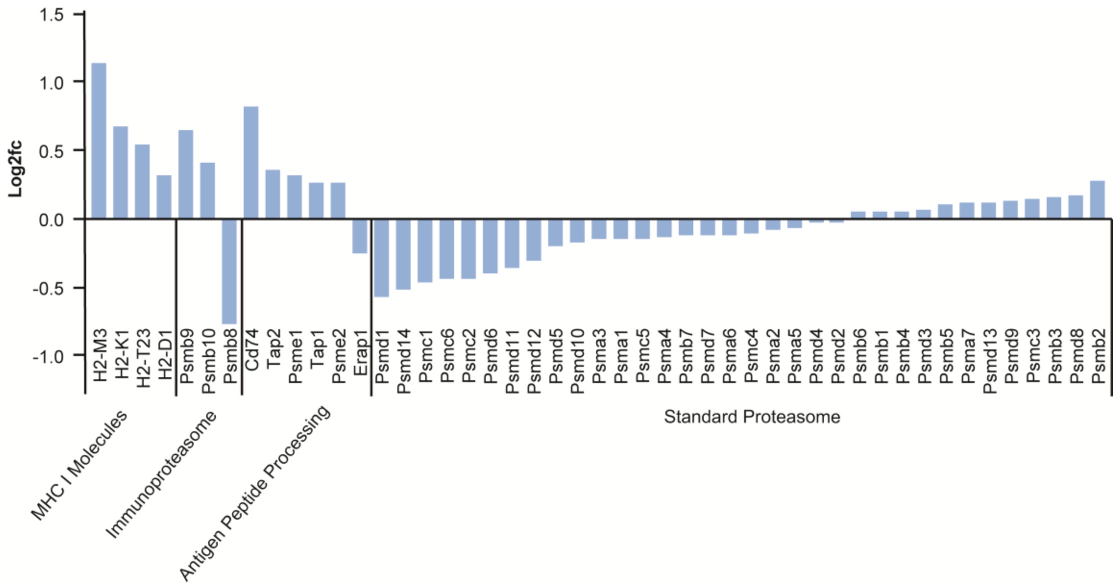

Supplemental figure 3. IRF7 S-nitrosylation down-regulates antigen presentation process in melanoma cells

The expression of immunoproteasome genes (Psmb9 and Psmb10) and antigen presentation and processing downstream genes (Psme1, Psme2, Tap1, Tap2, and Cd74) was increased by IRF7-C435A, while genes related to the standard proteasome (Psmd1, Psmd14, Psmc1, Psmc6, Psmc2, etc.) were decreased.

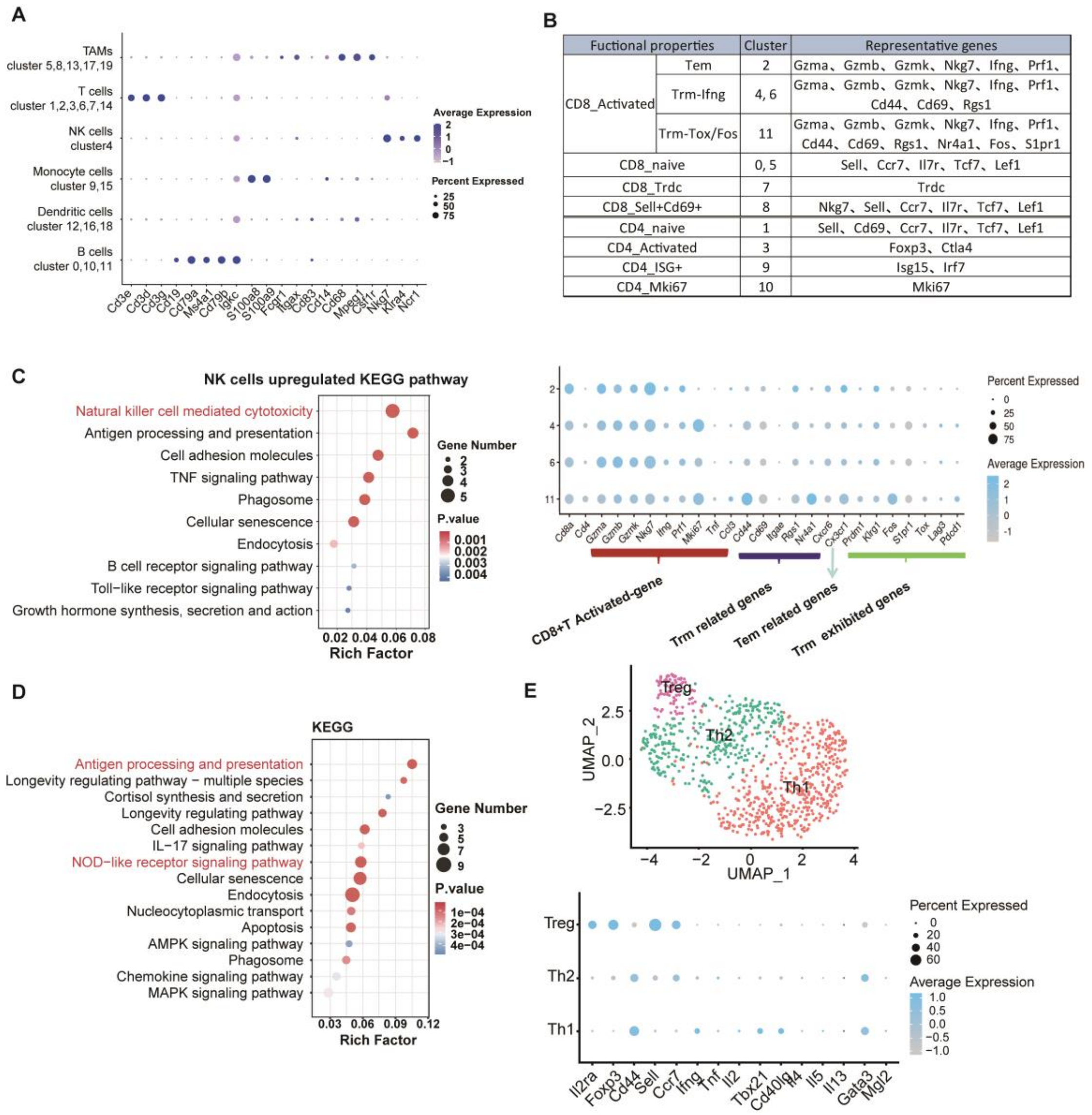

Fuctional properties		Cluster	Representative genes
CD8_Activated	Tem	2	Gzma、Gzmb、Gzmk、Nkg7、Ifng、Prf1、
	Trm-Ifng	4, 6	Gzma、Gzmb、Gzmk、Nkg7、Ifng、Prf1、Cd44、Cd69、Rgs1
	Trm-Tox/Fos	11	Gzma、Gzmb、Gzmk、Nkg7、Ifng、Prf1、Cd44、Cd69、Rgs1、Nr4a1、Fos、S1pr1
CD8_naive		0, 5	Sell、Ccr7、Il7r、Tcf7、Lef1
CD8_Trdc		7	Trdc
CD8_Sell+Cd69+		8	Nkg7、Sell、Ccr7、Il7r、Tcf7、Lef1
CD4_naive		1	Sell、Cd69、Ccr7、Il7r、Tcf7、Lef1
CD4_Activated		3	Foxp3、Ctla4
CD4_ISG+		9	Isg15、Irf7
CD4_Mki67		10	Mki67

Supplemental figure 4. B16-IRF7MUT reverses immune suppression in the tumor micro-environment

(A) Bubble heatmap illustrating the expression levels of specific marker genes in the CD45+ subsets.

(B) A list of signature genes expressed in different clusters of T lymphocyte subsets .

(C) KEGG analyses of 400 upregulated genes (fold change >1.2) in NK cells. The rich factor indicates the number of significant genes associated with the KEGG term divided by the total number of genes in the related pathway in the database

(D) KEGG analyses of 194 upregulated genes (fold change >1.2) in Dendritic cells. The rich factor indicates the number of signifcant genes associated with the KEGG term divided by the total number of genes in the related pathway in the database.

(E) Clustering of CD4_Activated cells and visualization of the subsets. (upper pannel)Uniform manifold approximation and projection (UMAP) analysis of single-cell RNA sequencing data revealed 3 cell types(Th1, Th2 and Treg) determined by 15 specifc markers (lower pannel).

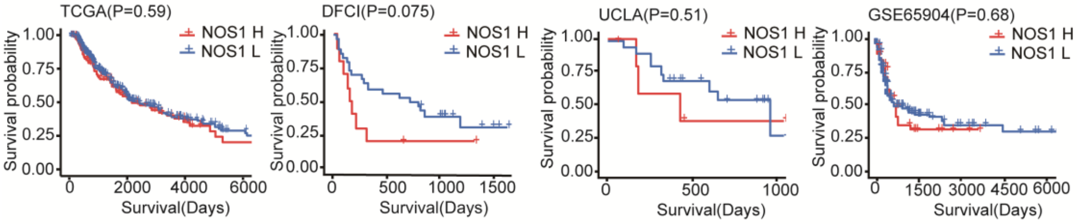

Supplemental figure 5. Clinical correlations exist between NOS1 expression and tumor immunity

Kaplan–Meier analysis of overall survival in melanoma patients with high CD45 expression in both the TCGA cohort and the GEO cohort (GSE19234, DFCI, UCLA).
