# Supplementary material for: S-nitrosylation of IRF7 induced by NOS1 expression in melanoma suppresses anti-tumor immunity
Source: Cell Death Dis. 2026 Jan 14;17(1):33. doi: 10.1038/s41419-025-08201-y (PMC12804790; doi:10.1038/s41419-025-08201-y)

Western bloting
Figure1E-left
GAPDH

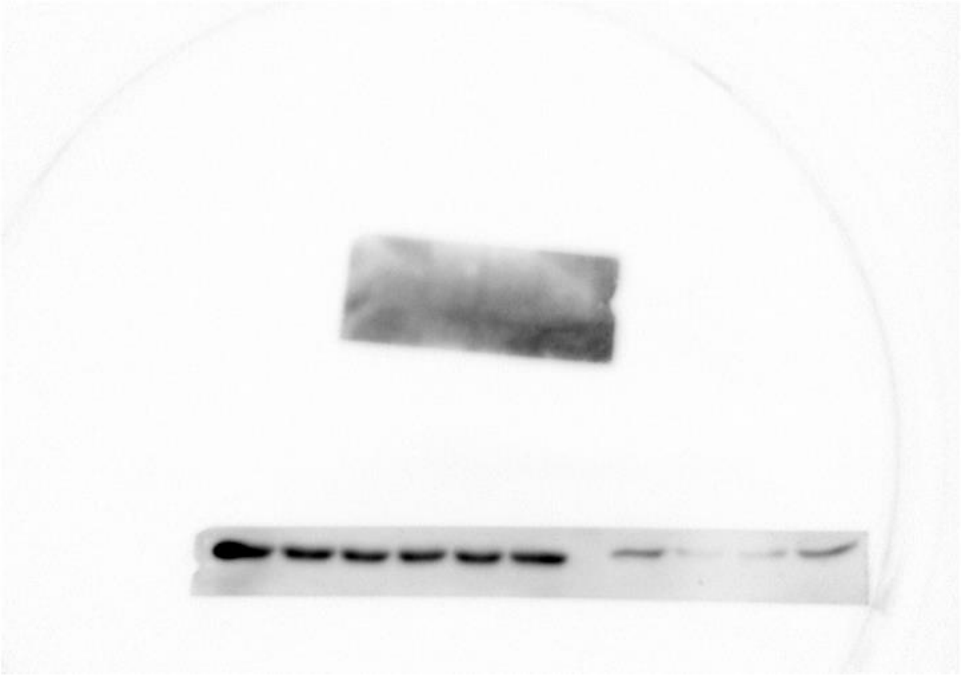

IRF7

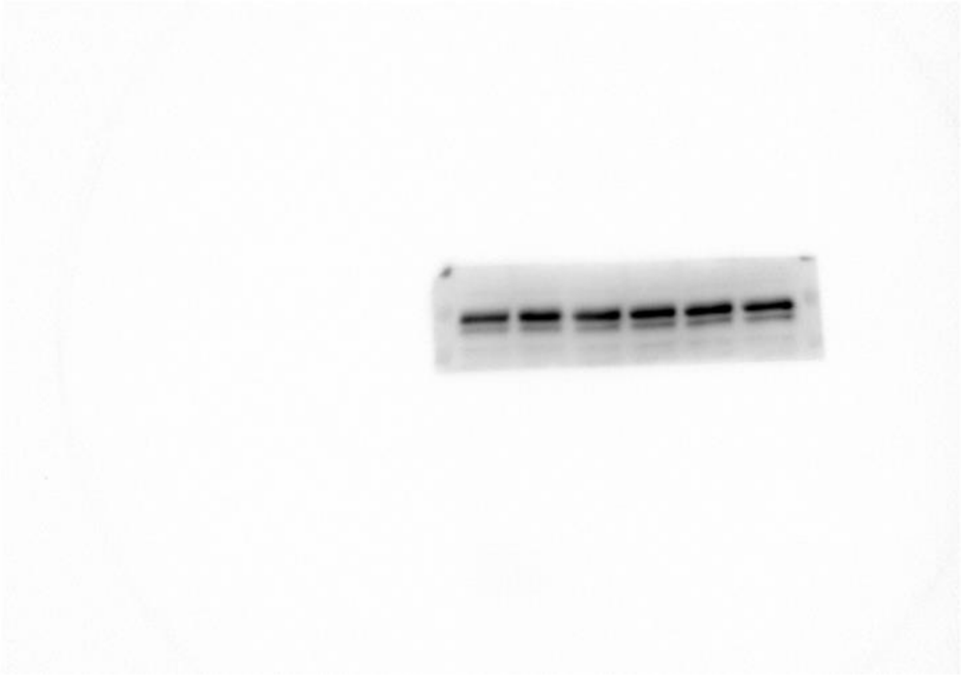

NOS1

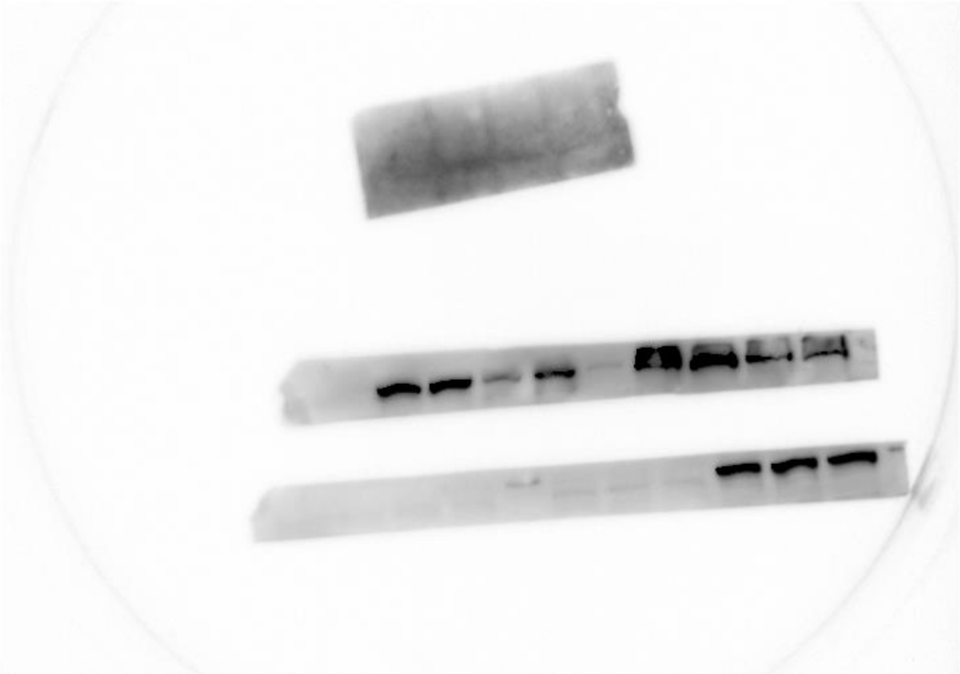

P-IRF7

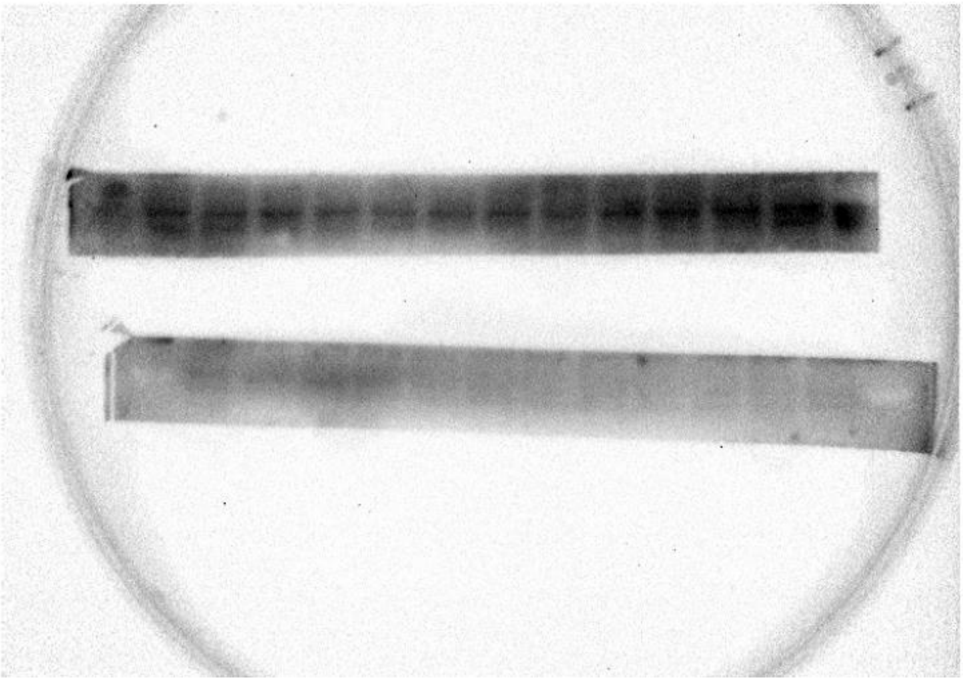

Figure1E-RIGHT

β-ACTIN

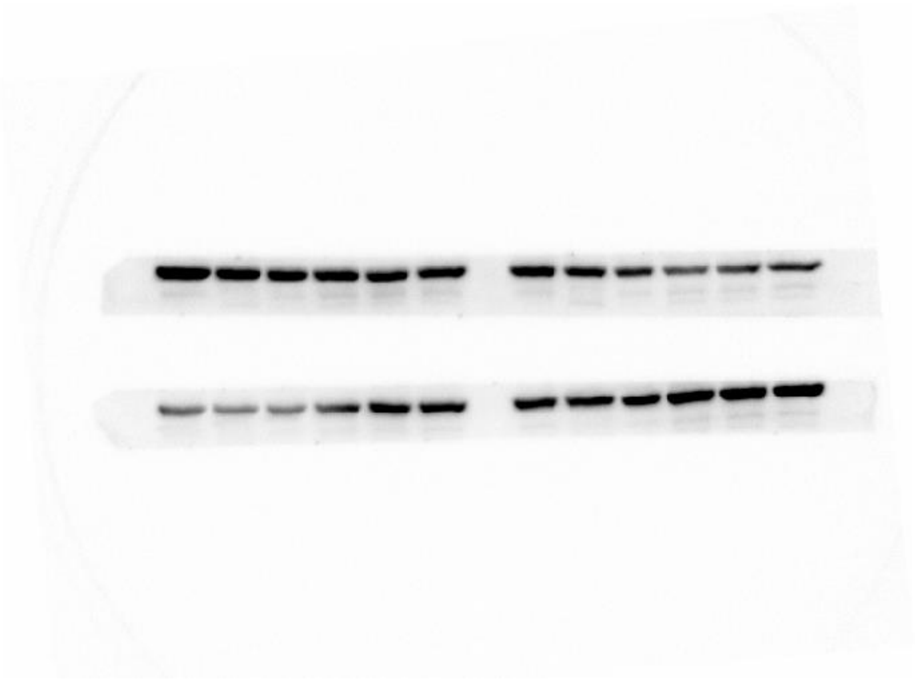

GAPDH

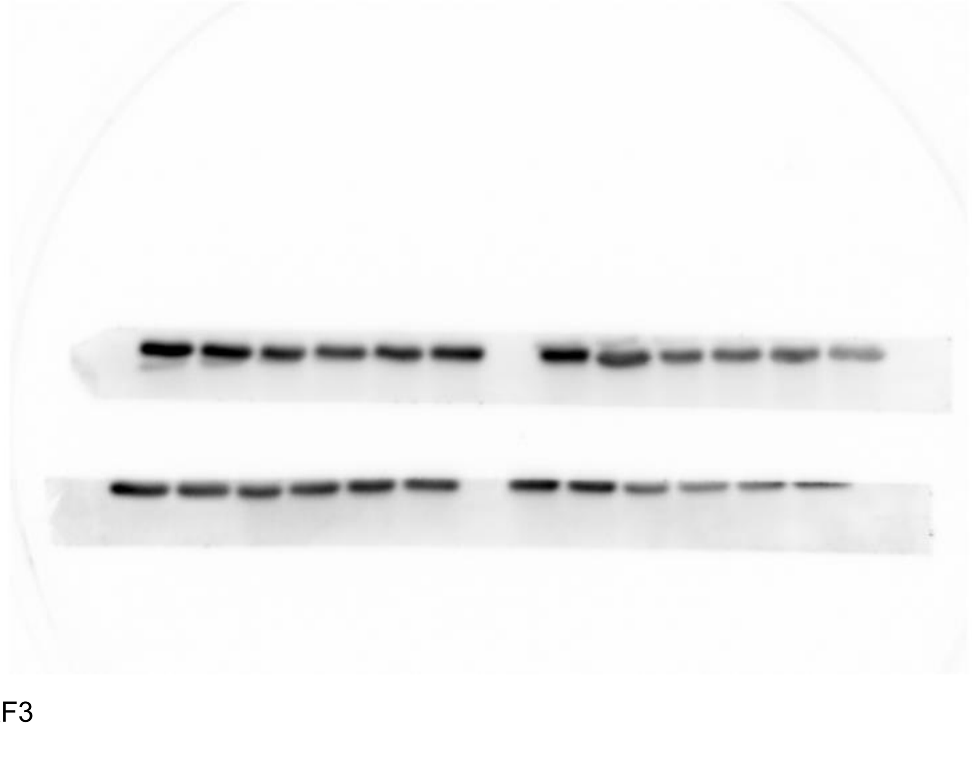

IRF3

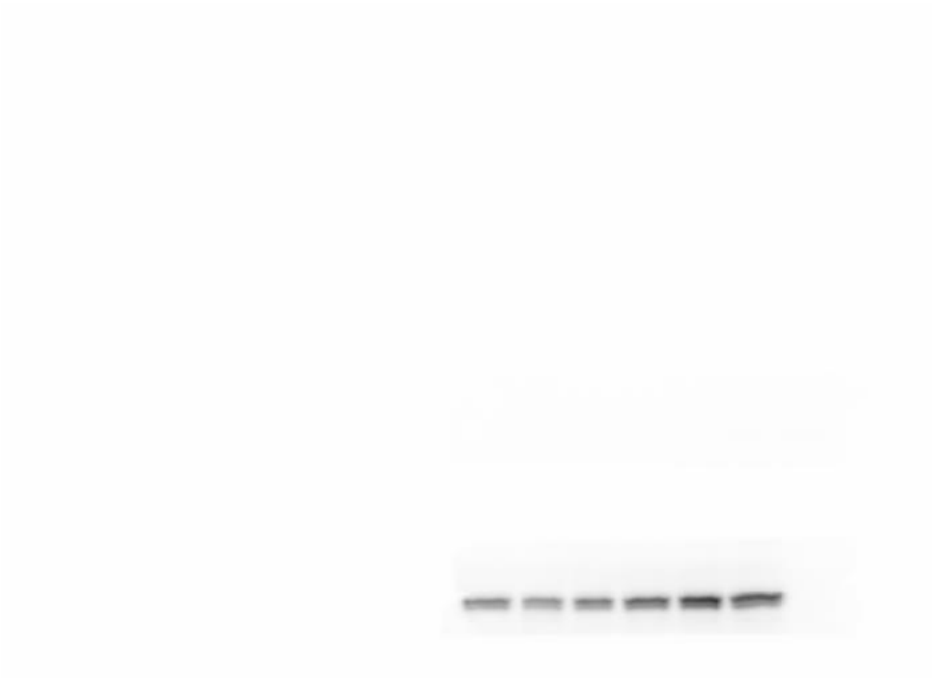

NOS1

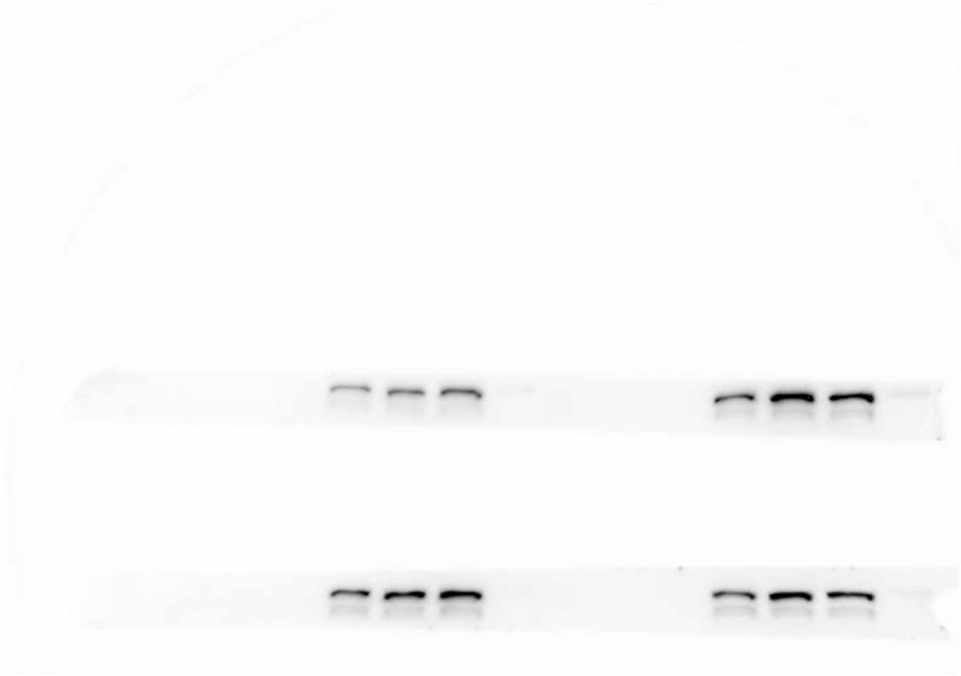

P-IRF3

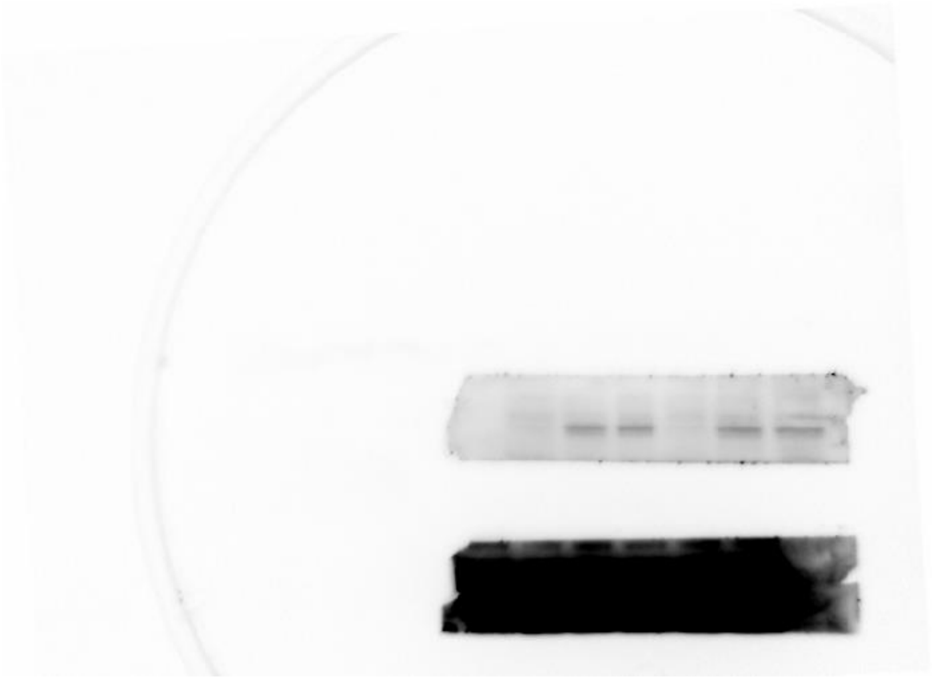

Figure1F-left

GAPDH

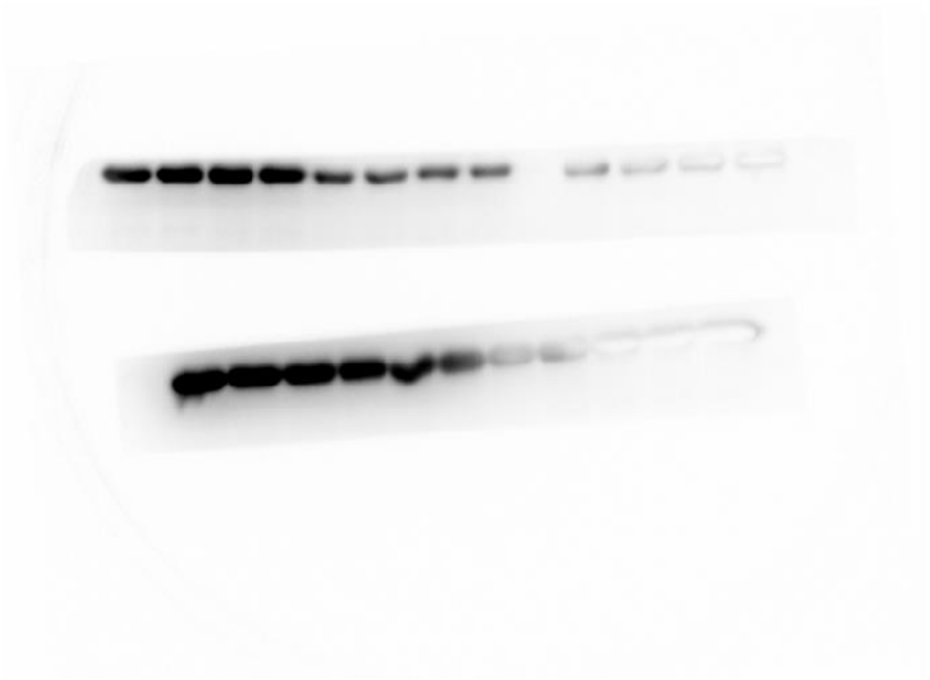

Input-IRF3

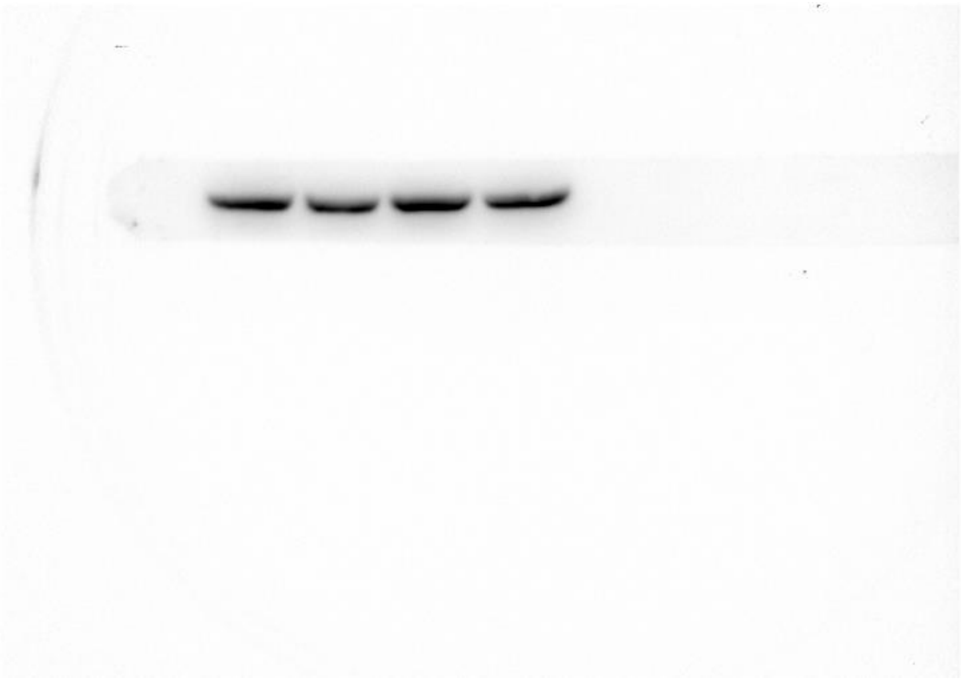

Input-IRF7

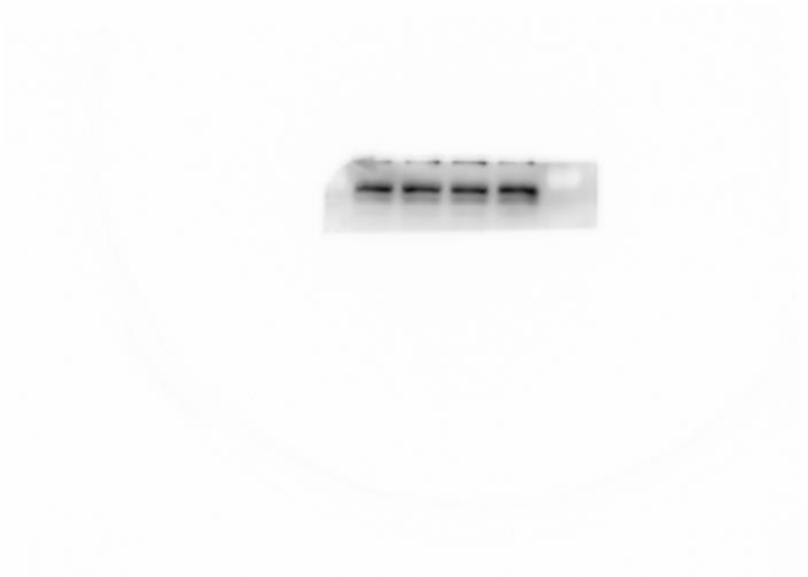

IP-IRF3

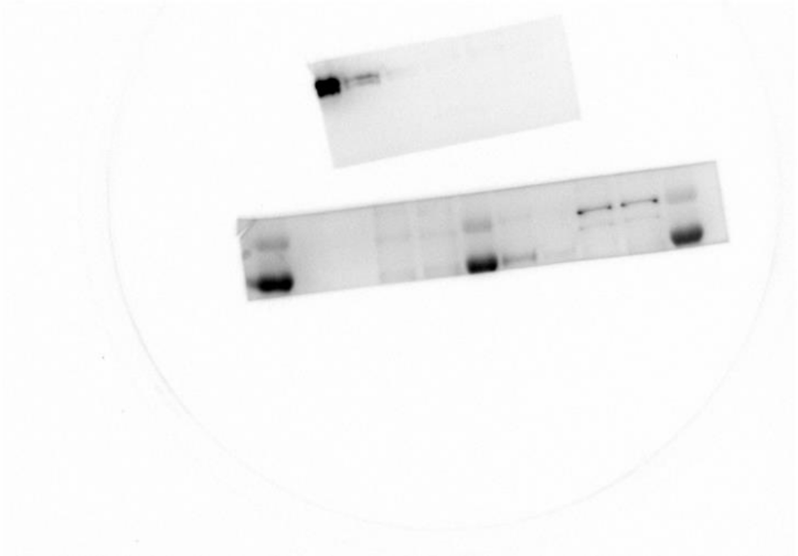

IP-IRF7

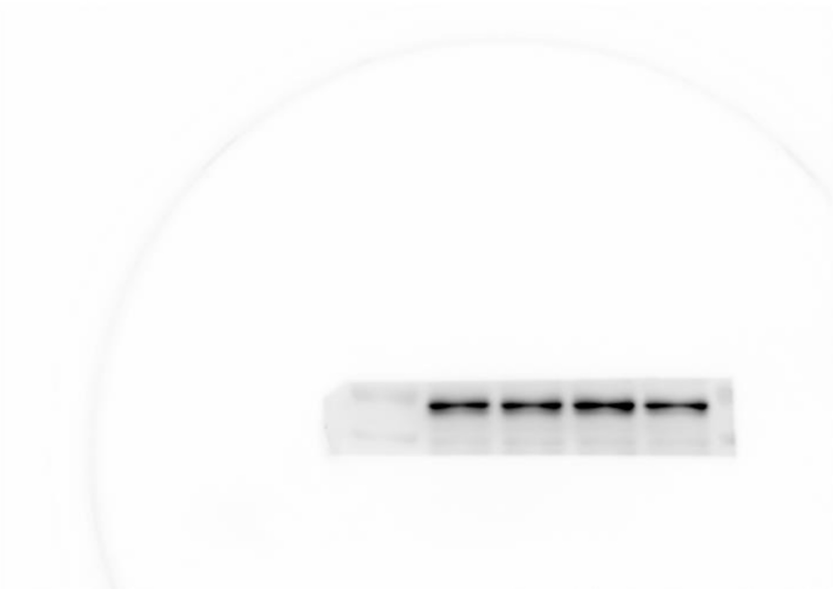

Figure1E-right

GAPDH

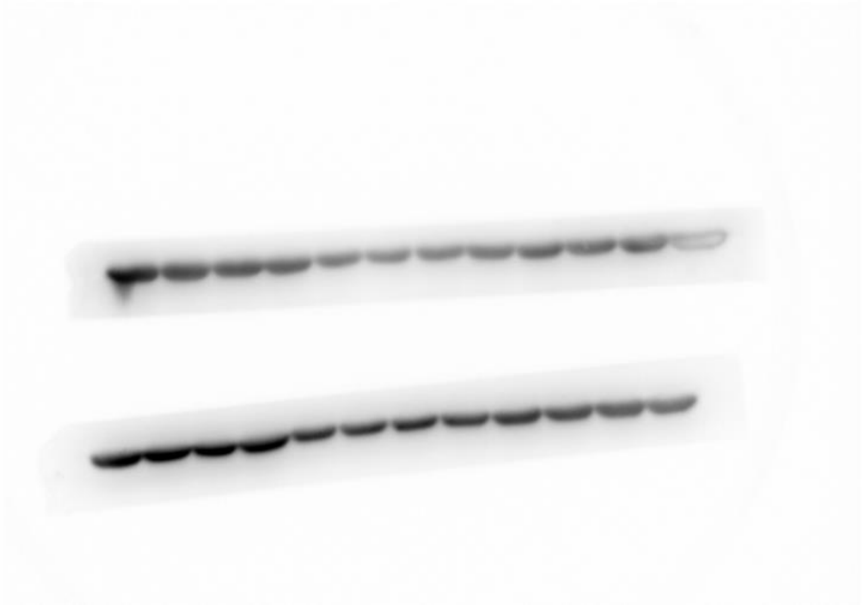

Input-IRF3

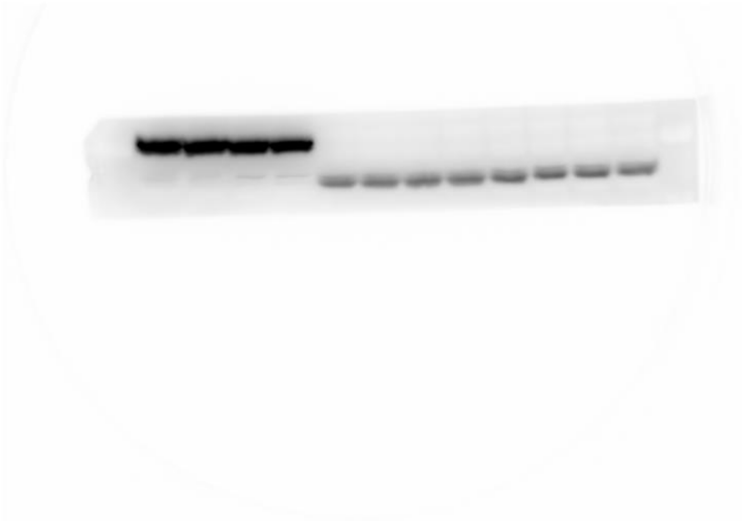

Input-IRF7

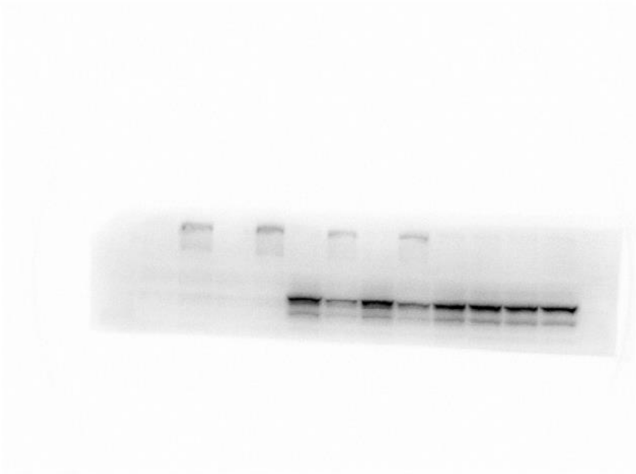

IP-IRF3

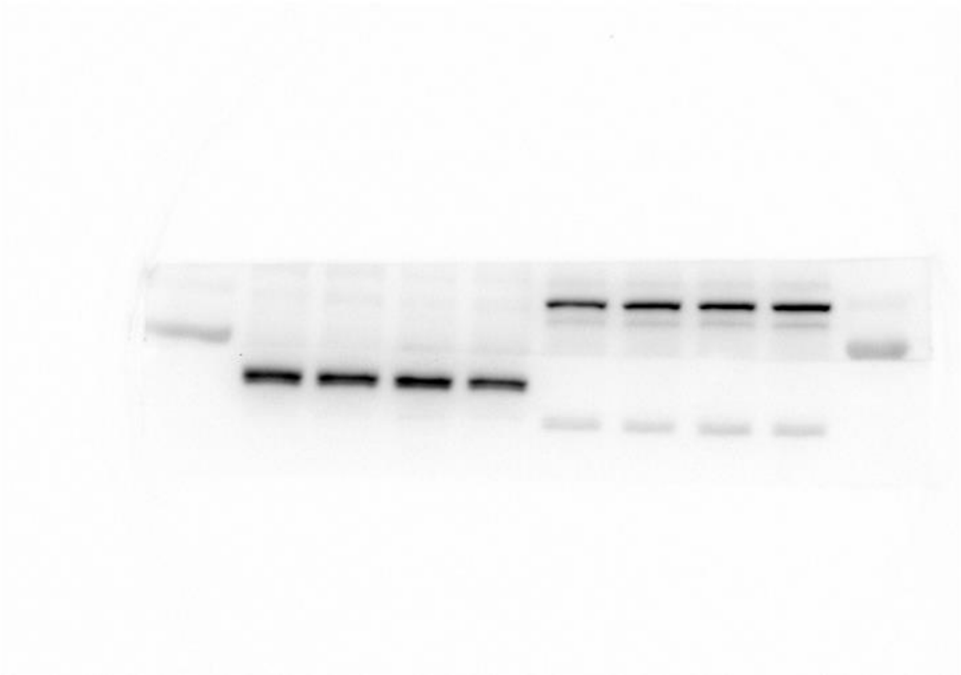

IP-IRF7

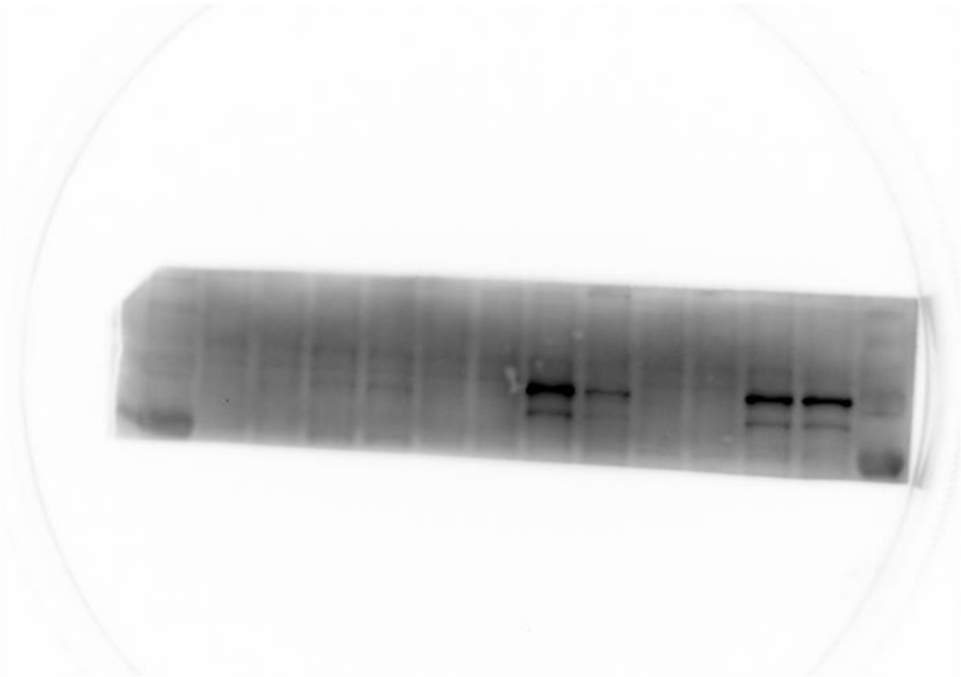

Figure1G

β-ACTIN

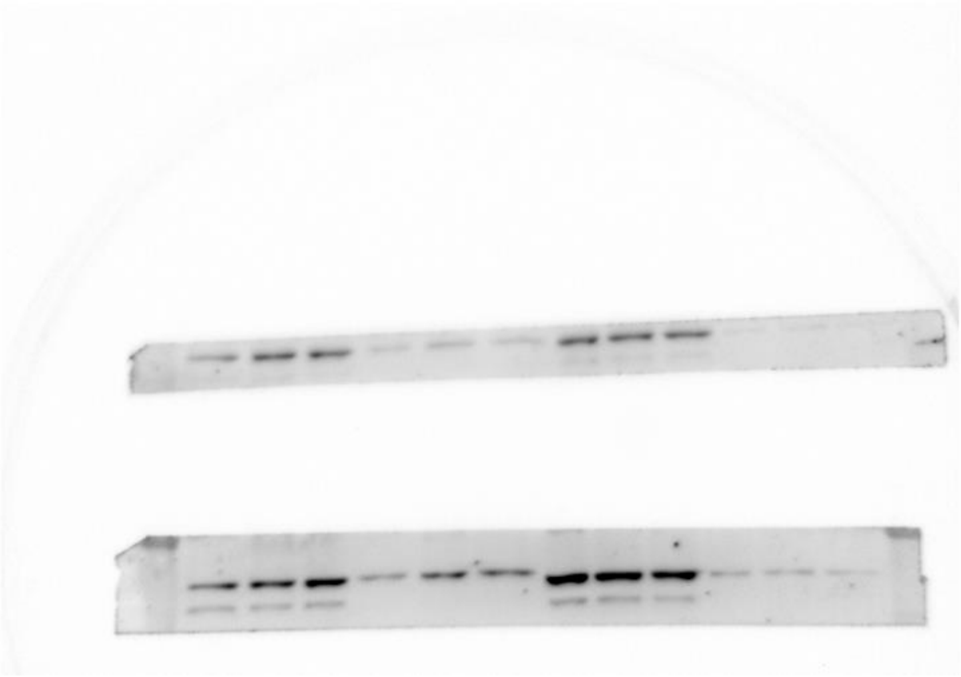

GAPDH

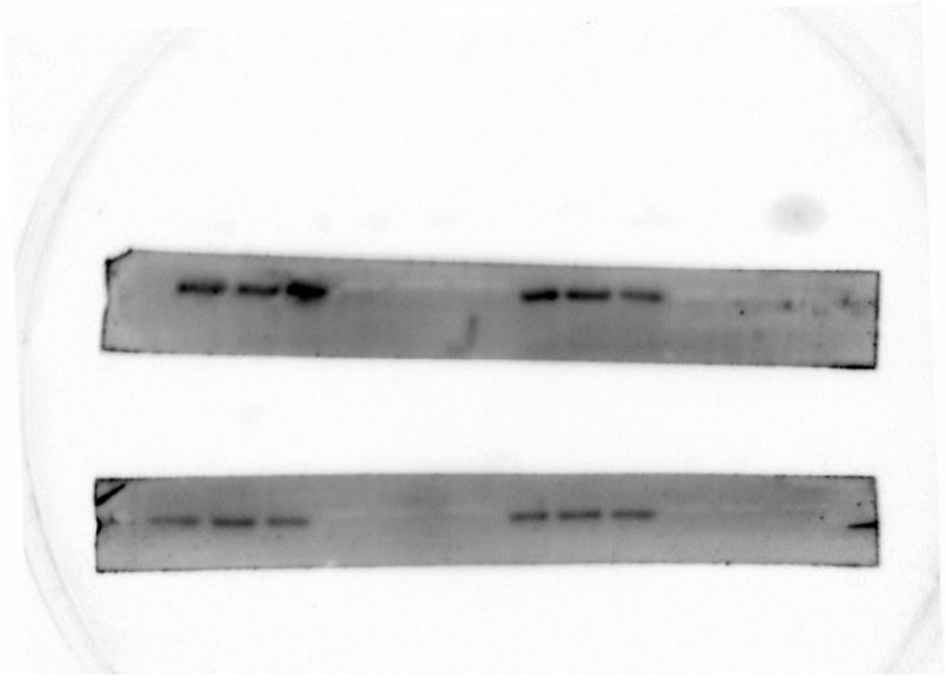

IRF3

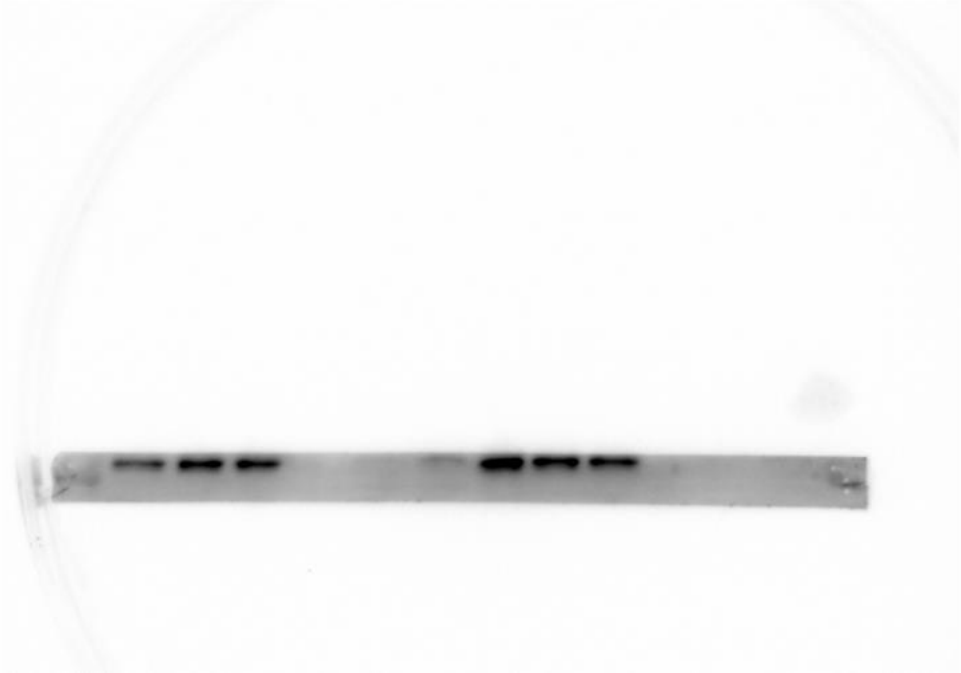

IRF7

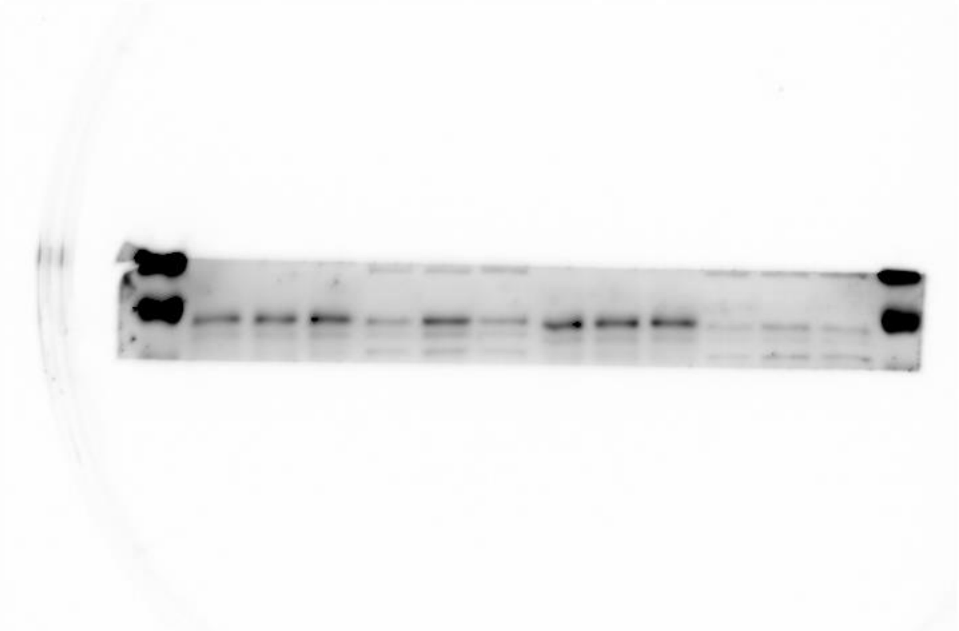

LAMB1

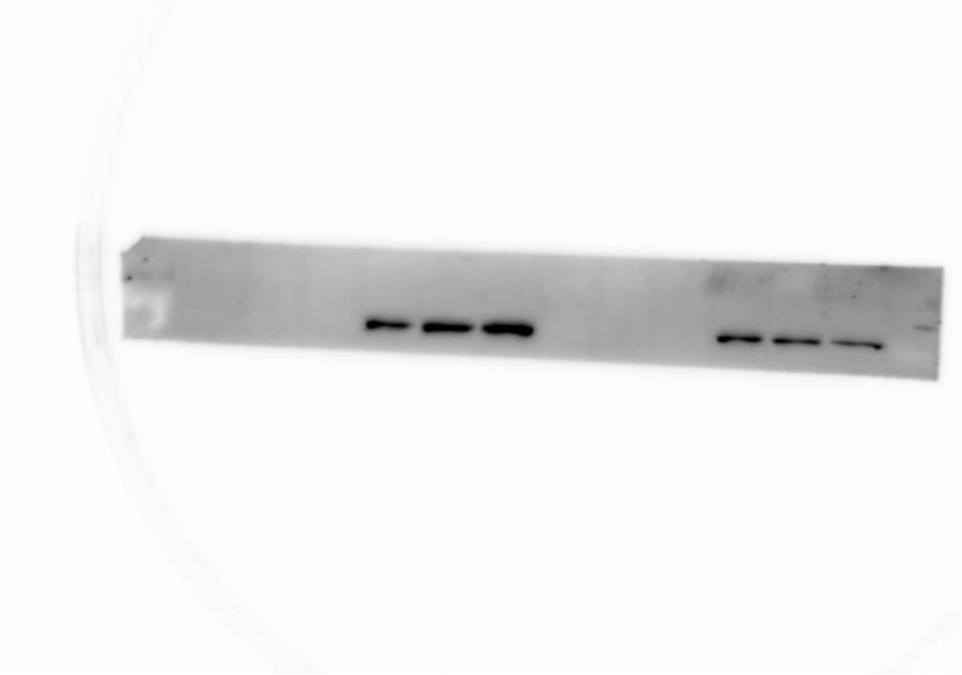

NOS1

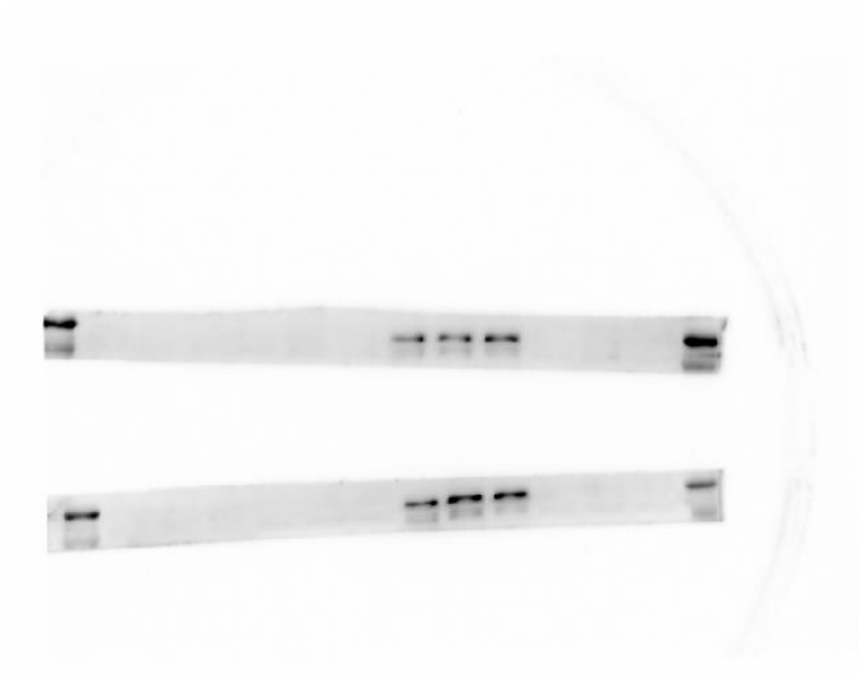

Figure1H

β -ACTIN

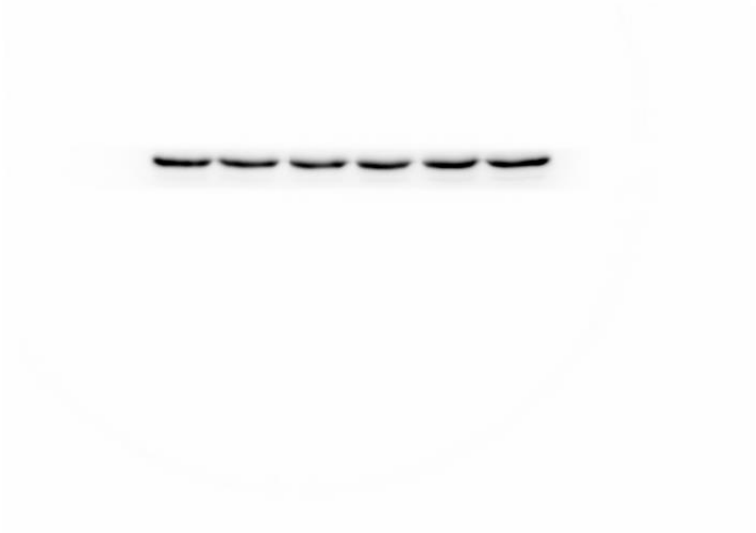

GAPDH

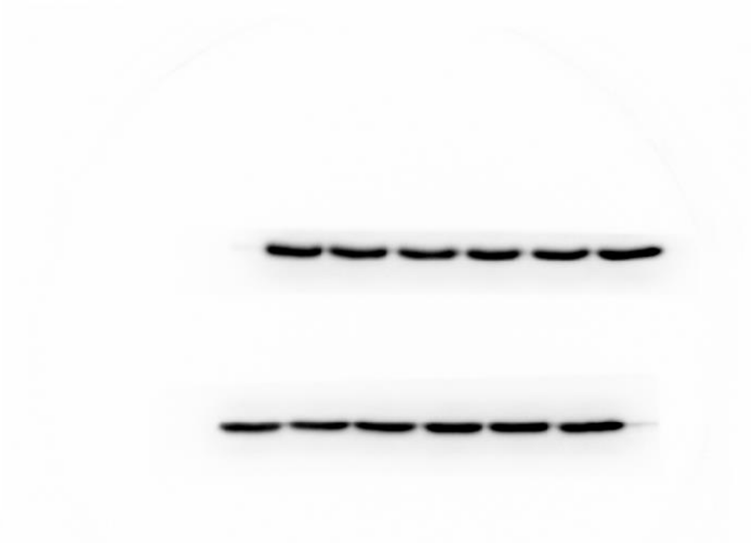

IRF3

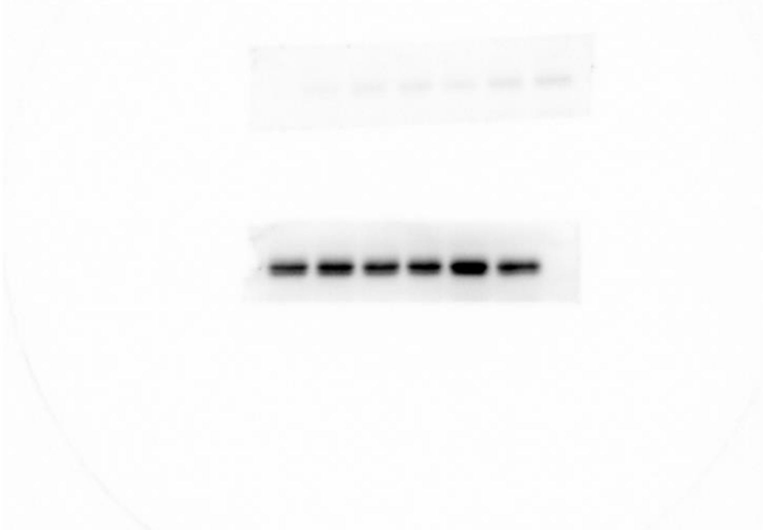

IRF7

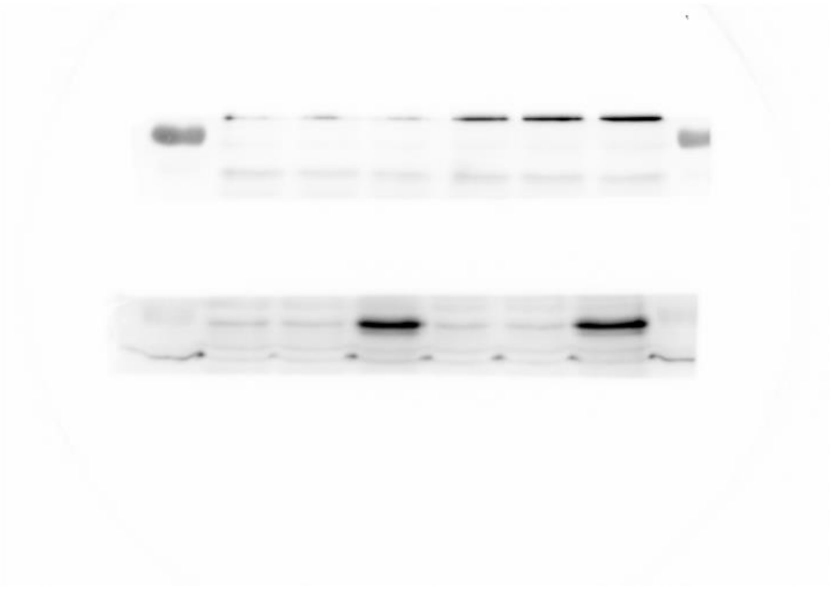

NOS1

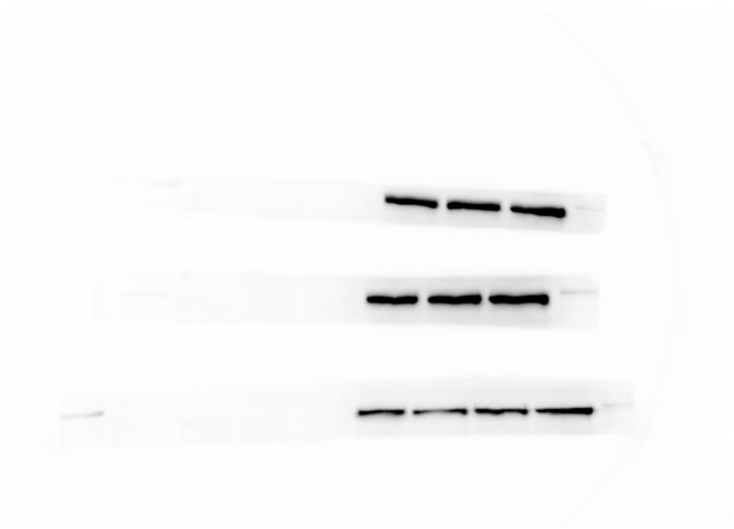

Figure2A-upper
GAPDH

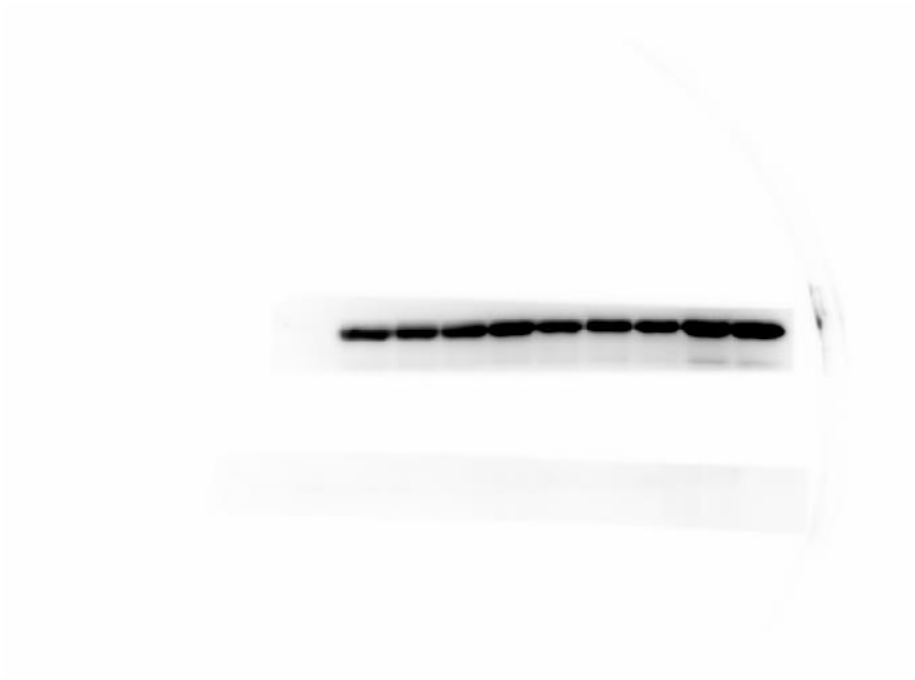

IRF7

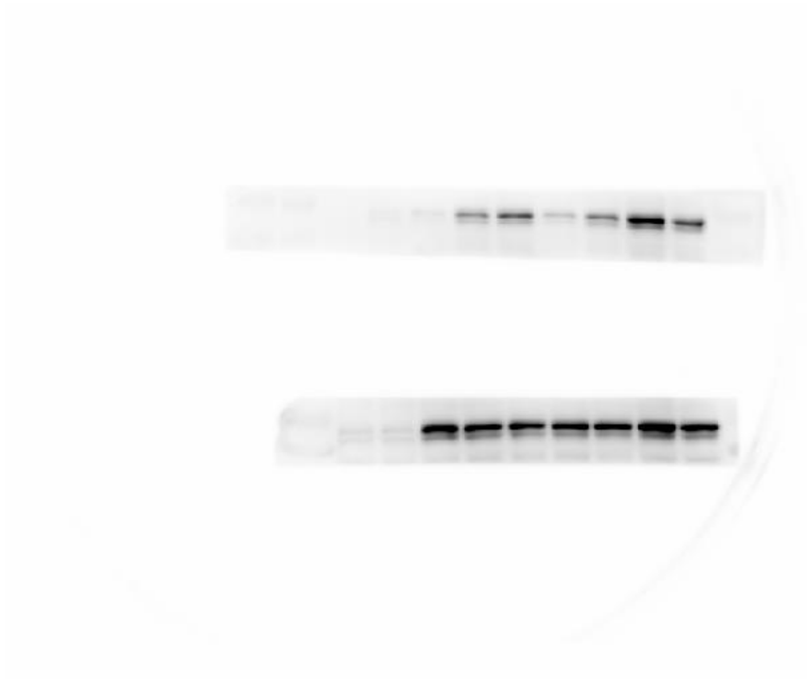

NOS1

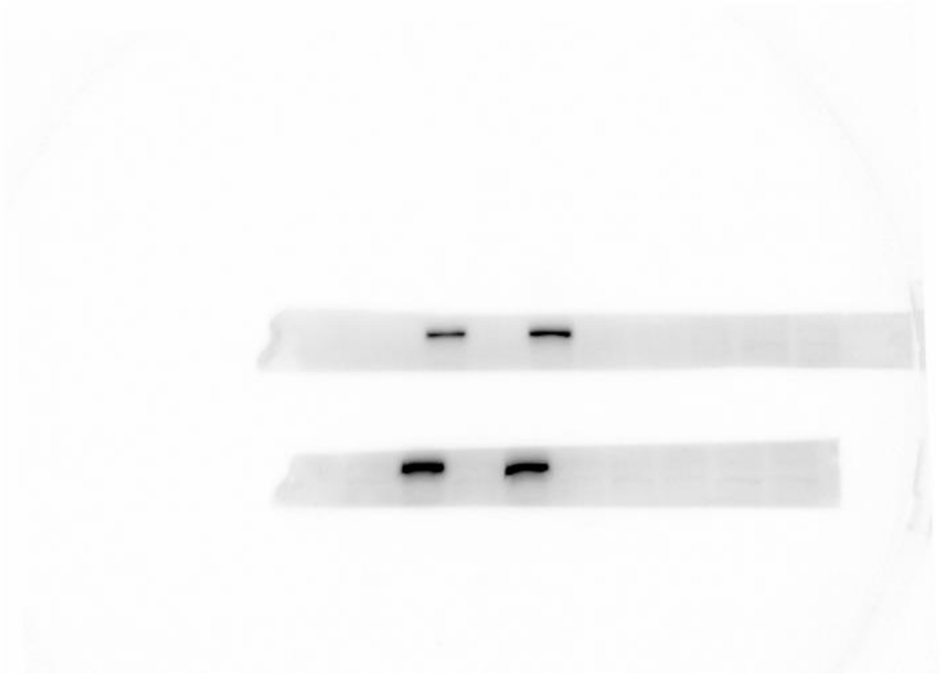

SNO-IRF7

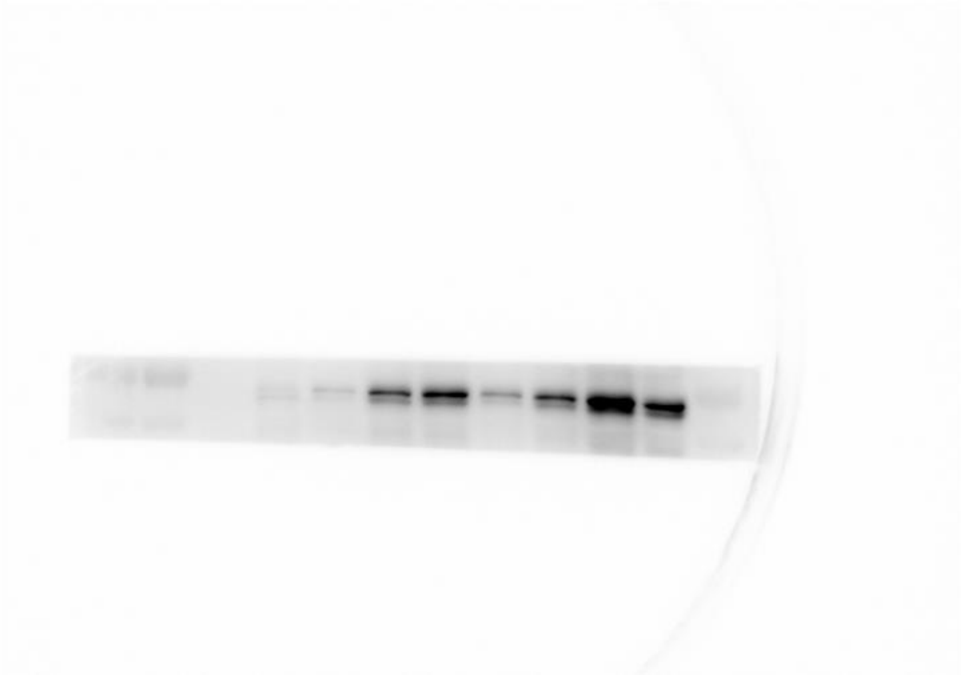

Figure2A-lower

GAPDH

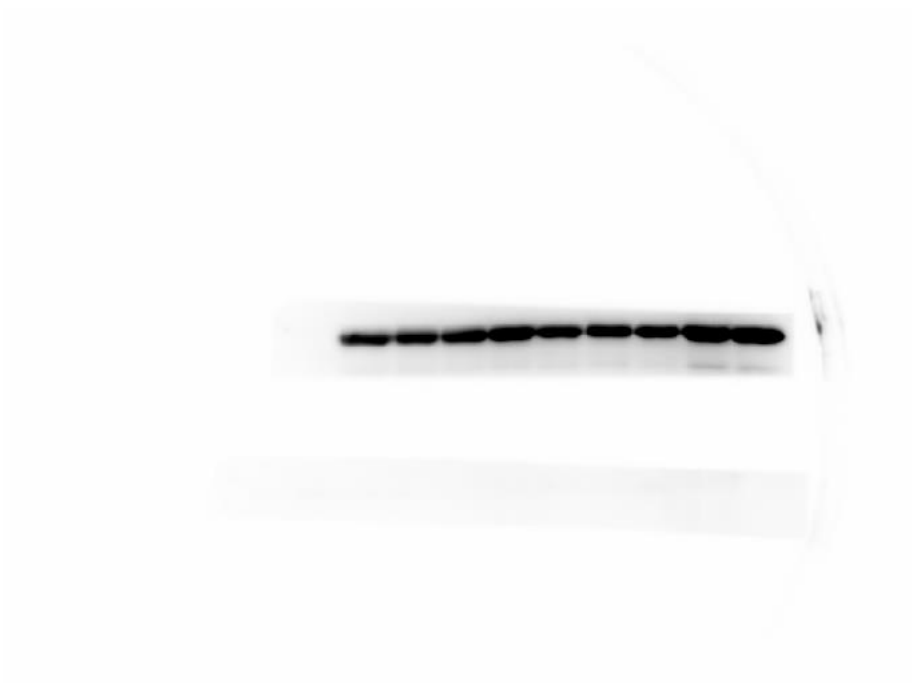

IRF7

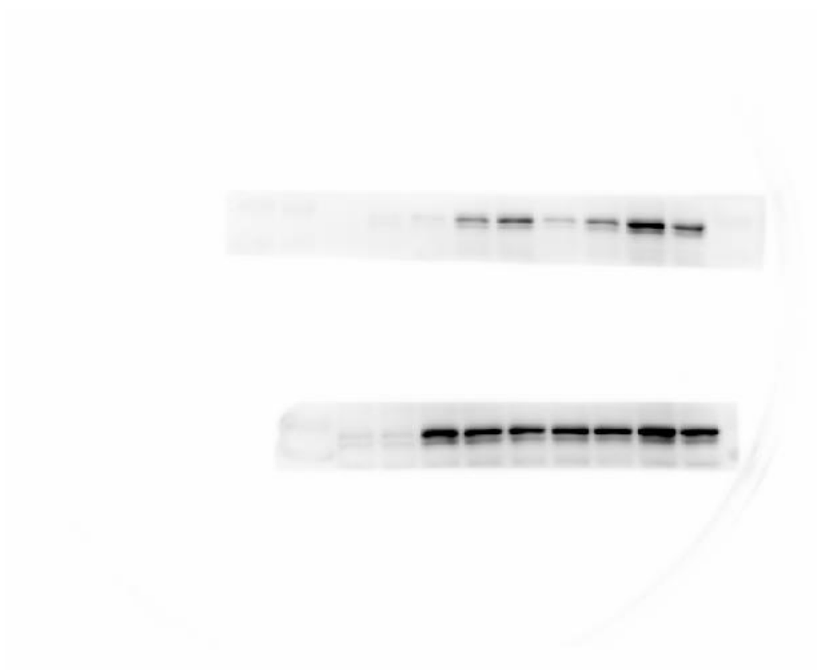

NOS1

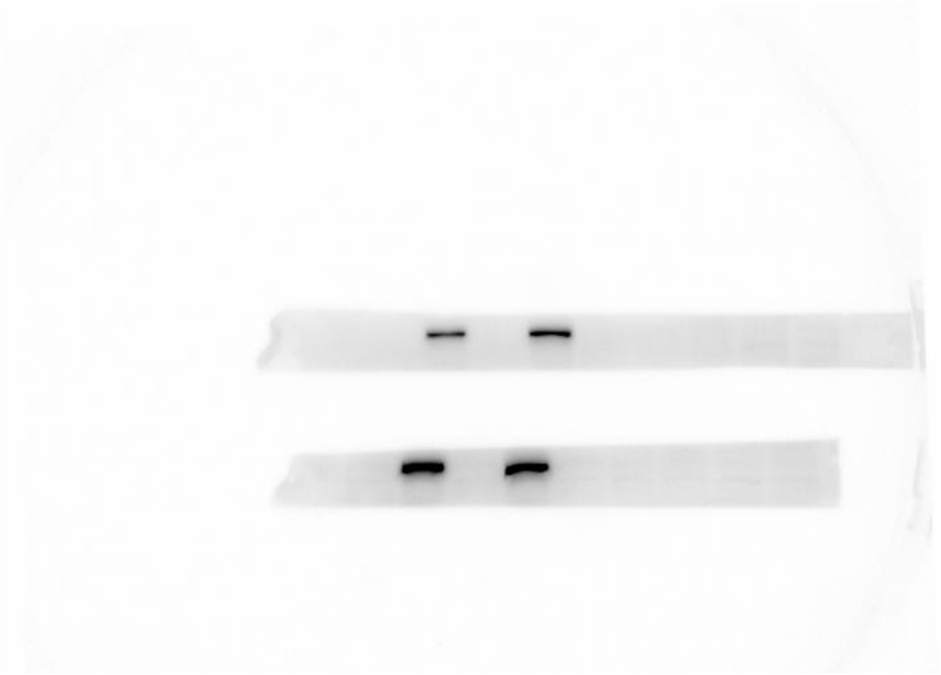

SNO-IRF7

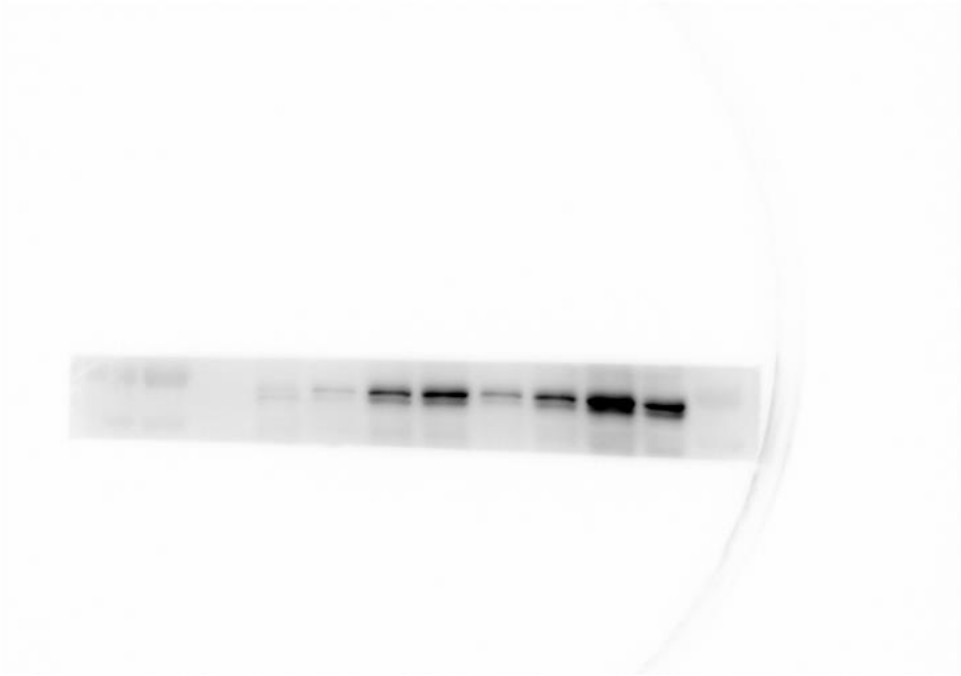

Figure2C-upper

GAPDH

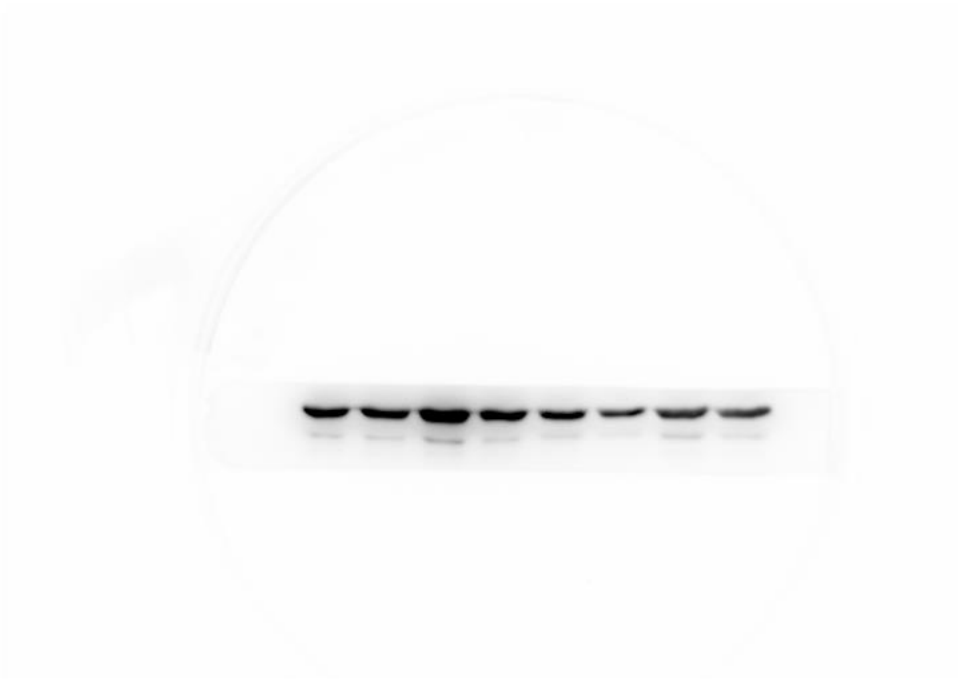

SNO-IRF7/FLAG

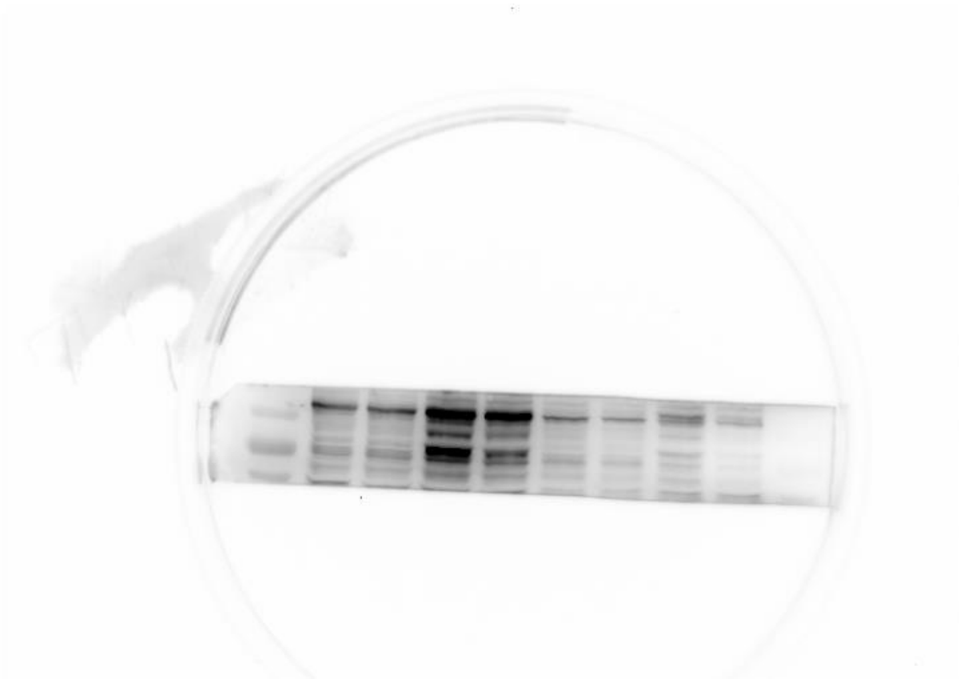

Figure2C-lower

Flag

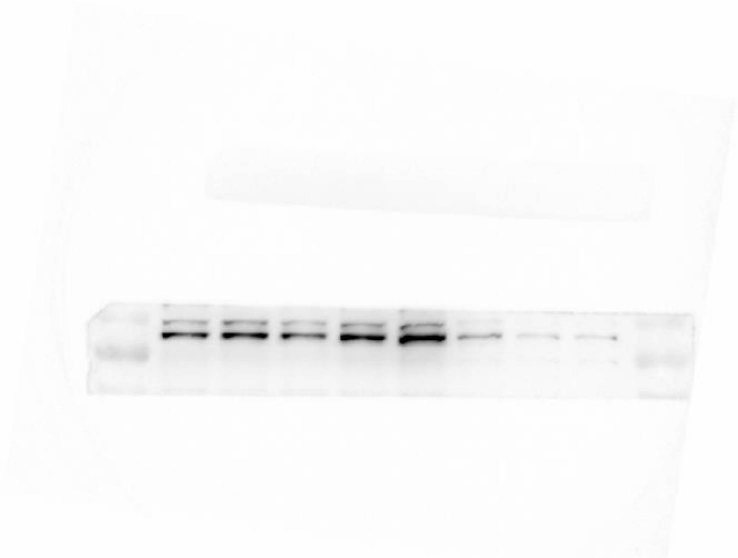

GAPDH

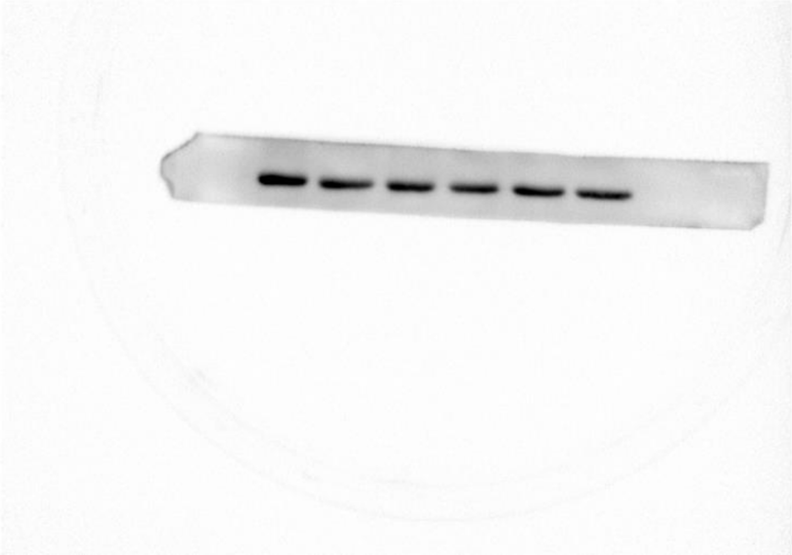

SNO-IRF7

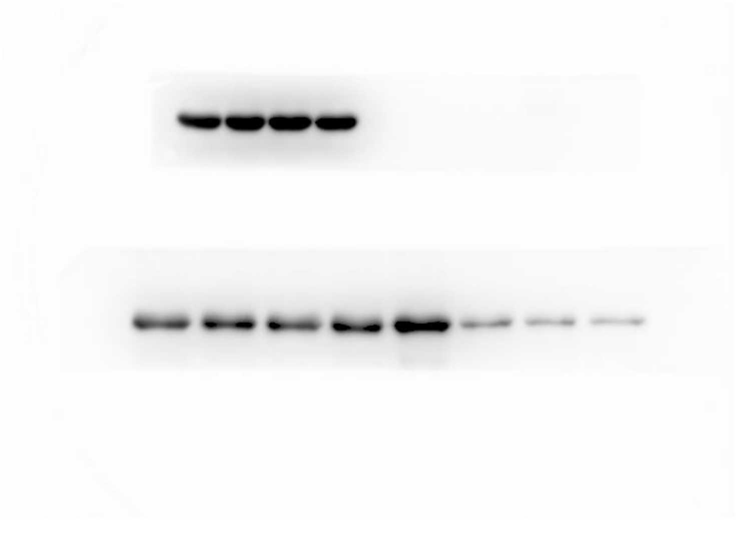

Supplemental figure1D-left

GAPDH

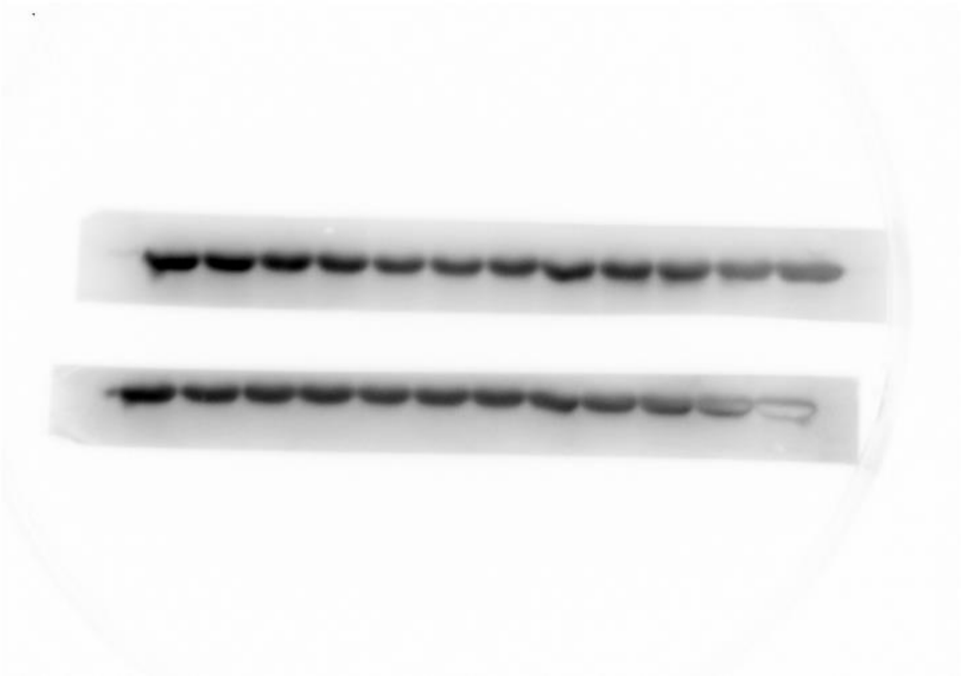

IRF7

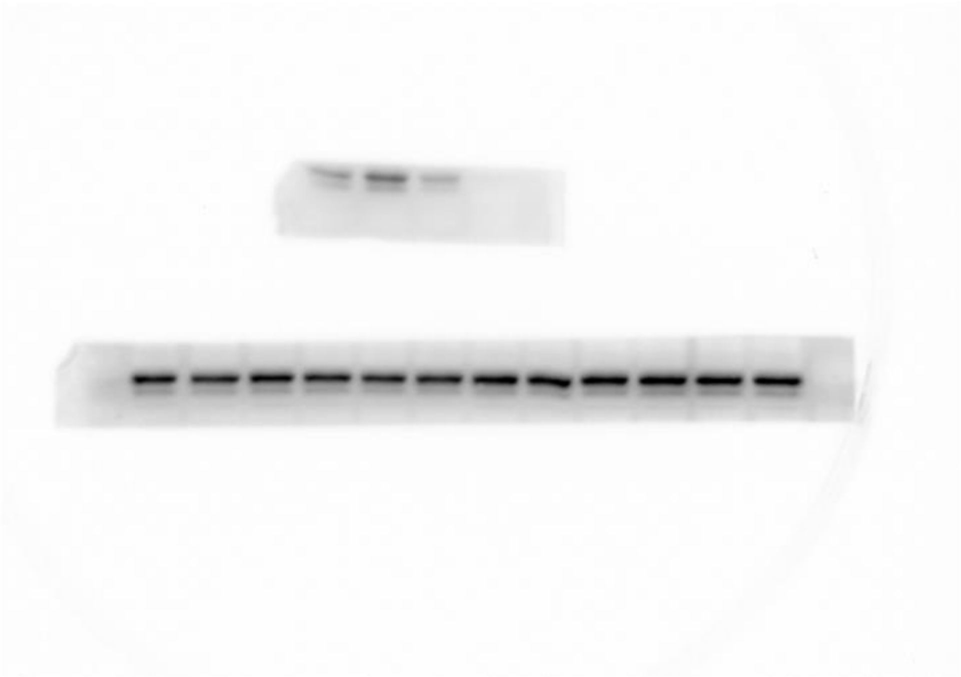

P-IRF7

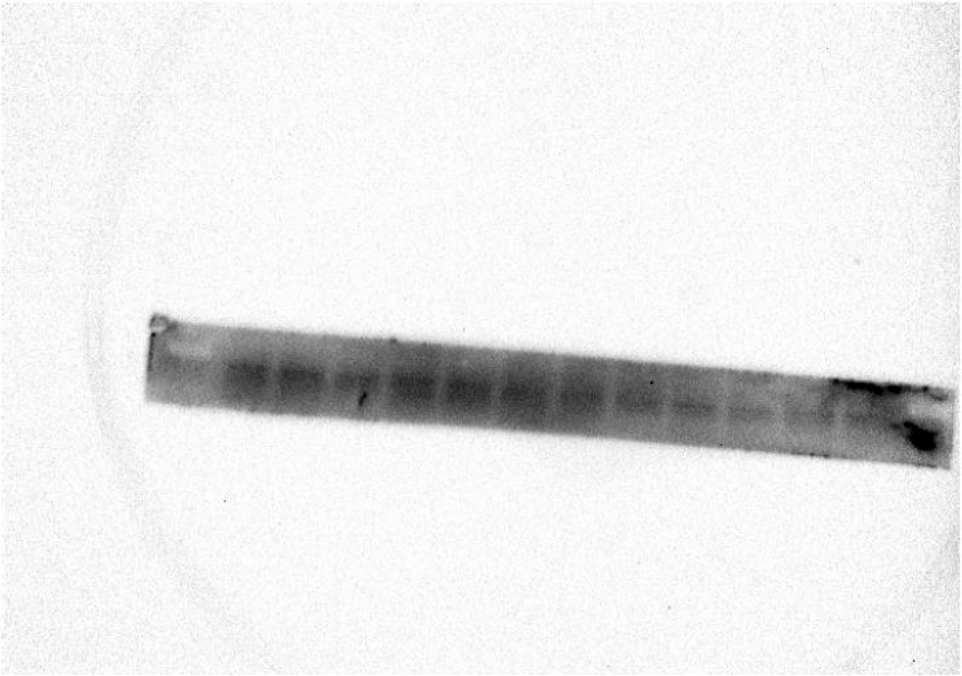

Supplemental figure1D-RIGHT

GAPDH

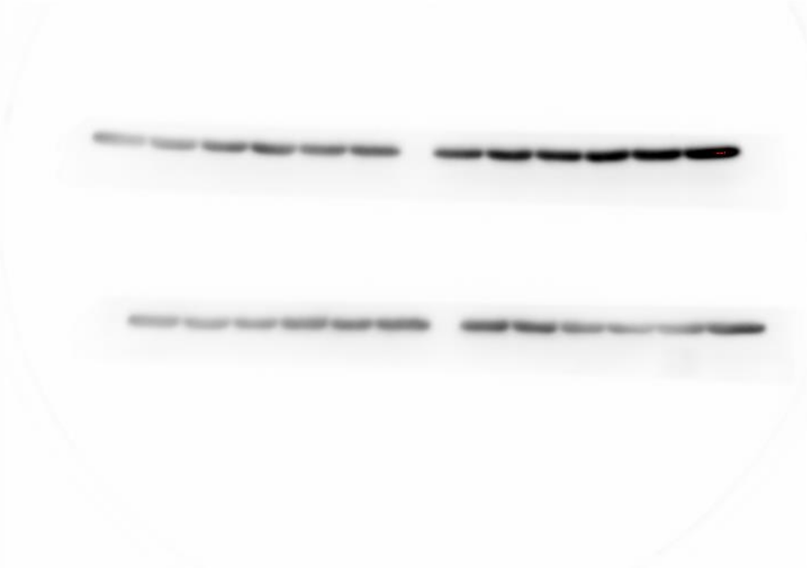

IRF7/P-IRF7

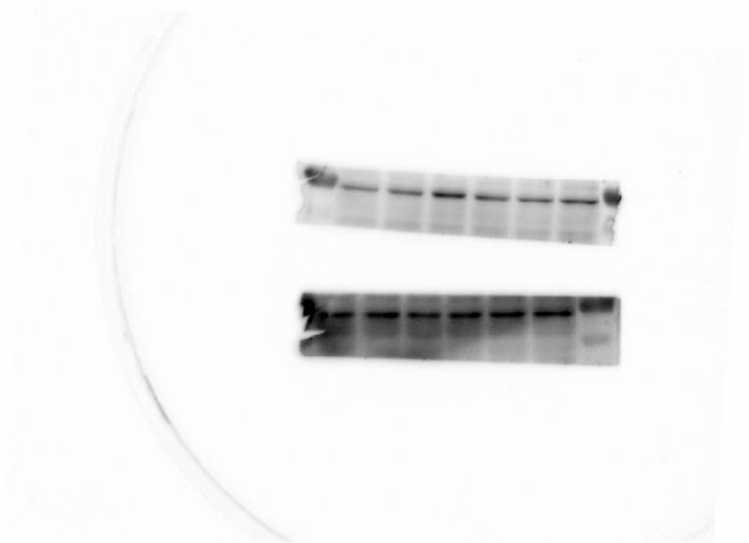

NOS1

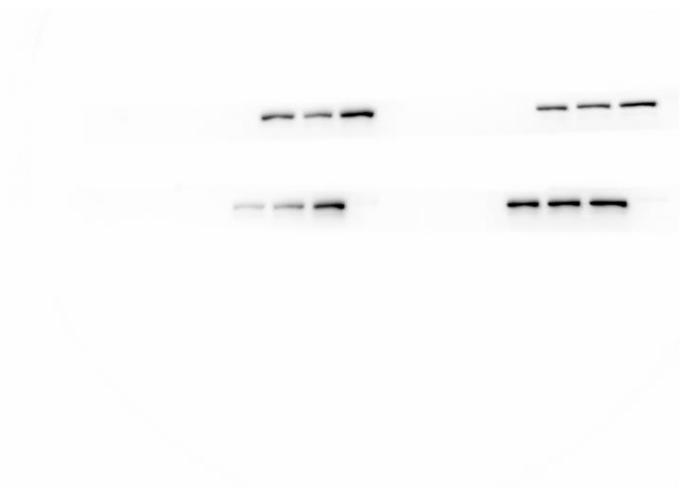

Supplemental figure1E

GAPDH

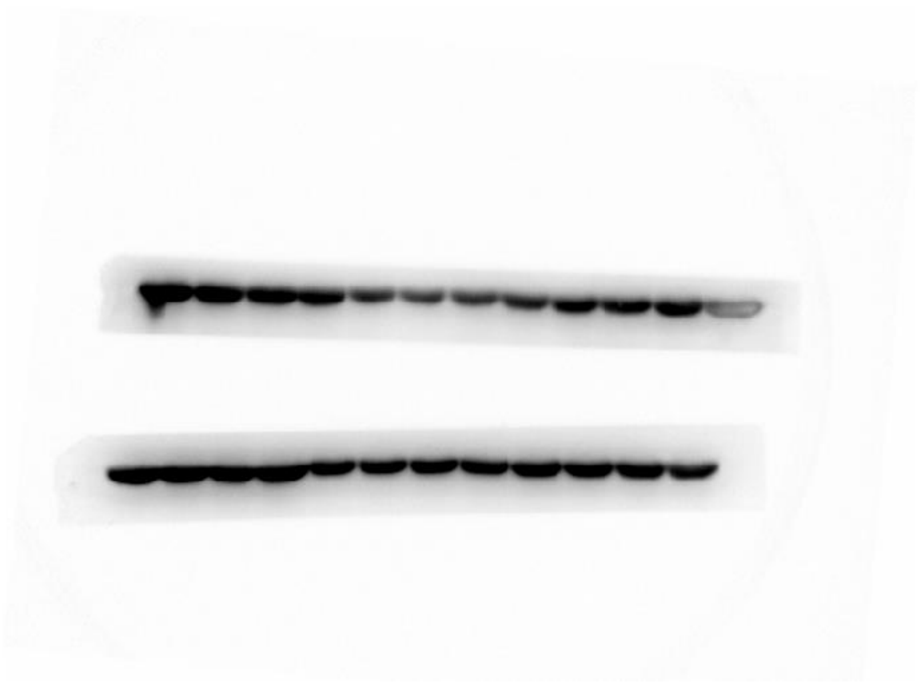

IRF7

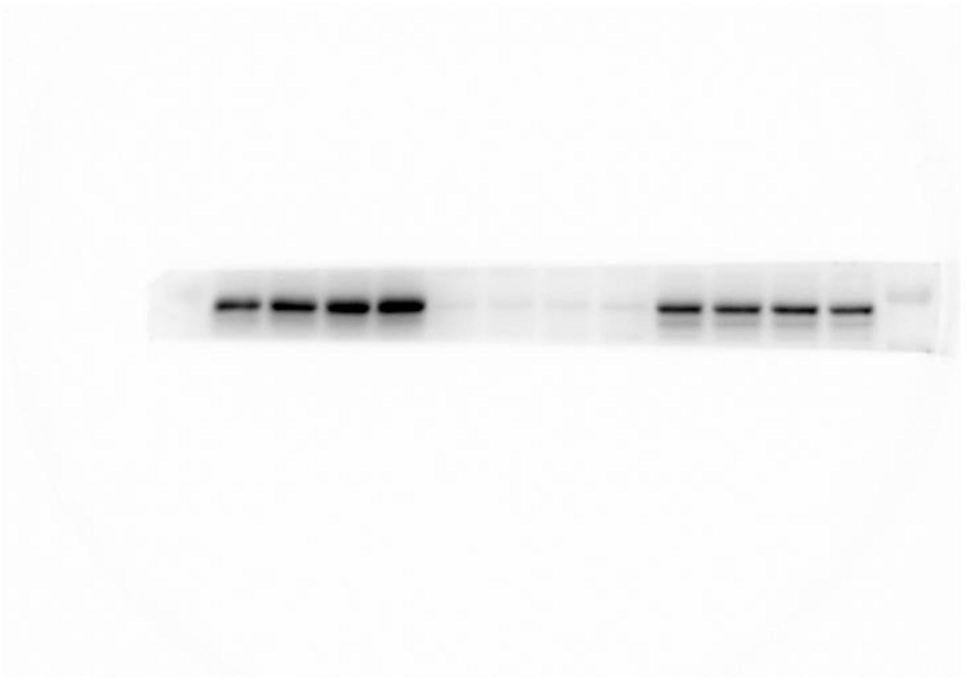

NOS1

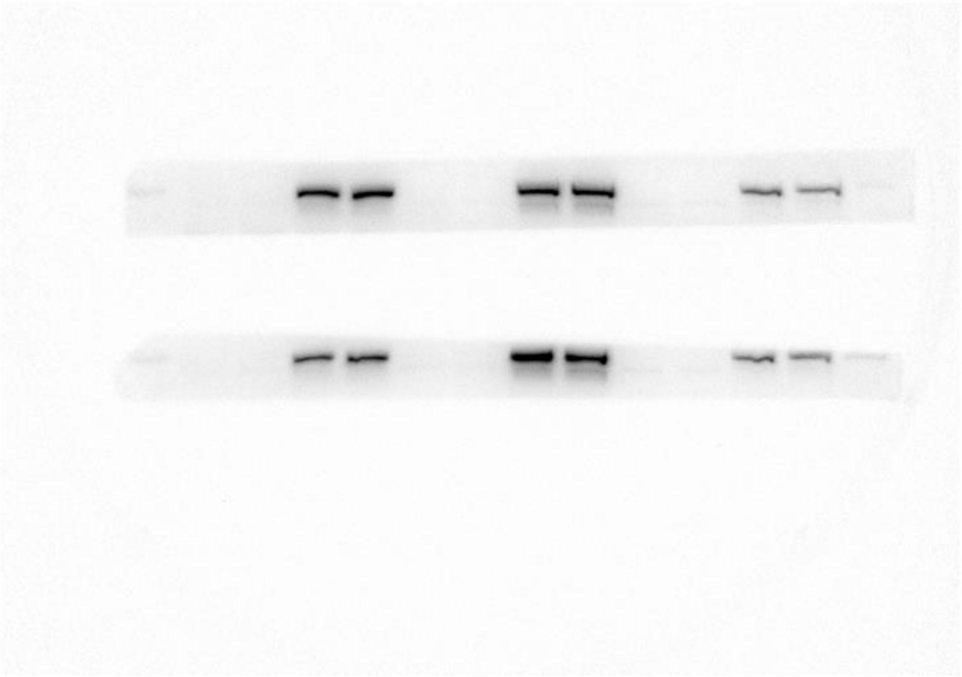

P-IRF7

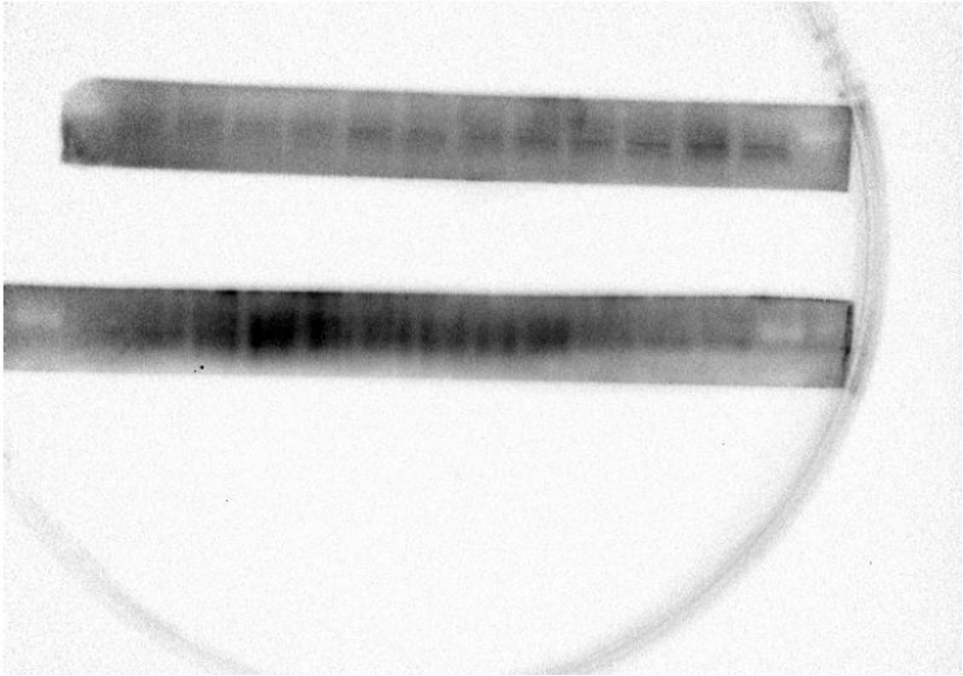

Supplement: Supplementary file 2 — western blots [file 41419_2025_8201_MOESM2_ESM.pdf]
